# CA10 regulates neurexin heparan sulfate addition via a direct binding in the secretory pathway

**DOI:** 10.15252/embr.202051349

**Published:** 2021-02-15

**Authors:** Laia Montoliu‐Gaya, Daniel Tietze, Debora Kaminski, Ekaterina Mirgorodskaya, Alesia A Tietze, Fredrik H Sterky

**Affiliations:** ^1^ Department of Laboratory Medicine Institute for Biomedicine Sahlgrenska Academy University of Gothenburg Gothenburg Sweden; ^2^ Wallenberg Centre for Molecular and Translational Medicine University of Gothenburg Gothenburg Sweden; ^3^ Department of Chemistry and Molecular Biology Faculty of Science University of Gothenburg Gothenburg Sweden; ^4^ Department of Clinical Chemistry Sahlgrenska University Hospital Gothenburg Sweden; ^5^ Proteomics Core Facility University of Gothenburg Gothenburg Sweden

**Keywords:** CA10, carbonic anhydrase‐related protein, heparan sulfate, neurexin, synapse, Membrane & Intracellular Transport, Neuroscience, Post-translational Modifications, Proteolysis & Proteomics

## Abstract

Neurexins are presynaptic adhesion molecules that shape the molecular composition of synapses. Diversification of neurexins in numerous isoforms is believed to confer synapse‐specific properties by engaging with distinct ligands. For example, a subset of neurexin molecules carry a heparan sulfate (HS) glycosaminoglycan that controls ligand binding, but how this post‐translational modification is controlled is not known. Here, we observe that CA10, a ligand to neurexin in the secretory pathway, regulates neurexin‐HS formation. CA10 is exclusively found on non‐HS neurexin and CA10 expressed in neurons is sufficient to suppress HS addition and attenuate ligand binding and synapse formation induced by ligands known to recruit HS. This effect is mediated by a direct interaction in the secretory pathway that blocks the primary step of HS biosynthesis: xylosylation of the serine residue. NMR reveals that CA10 engages residues on either side of the serine that can be HS‐modified, suggesting that CA10 sterically blocks xylosyltransferase access in Golgi. These results suggest a mechanism for the regulation of HS on neurexins and exemplify a new mechanism to regulate site‐specific glycosylations.

## Graphical abstract

CA10 binds the presynaptic adhesion molecules neurexin in the secretory pathway and inhibits their modification with heparan sulfate, thereby modifying the ligand‐binding properties of neurexin.

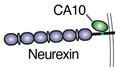

## Introduction

The flow of information through the circuits of our brain depends on synaptic connections that relay signals between interconnected neurons. While all synapses share a set of common features—such as a presynaptic vesicle release machinery juxtaposed to appropriate receptors on the postsynaptic membrane—structural and functional diversity allows for differential information processing and plasticity at the level of individual synapses (Grant & O'Dell, 2001; Sheng & Kim, 2011; Südhof, 2013). The properties of a synapse depend on the composition and organization of its molecular building blocks. Synaptic cell adhesion molecules and their trans‐synaptic interactions are believed to be major determinants of this molecular architecture by coordinating the recruitment and assembly of components on either side of the synaptic cleft (de Wit & Ghosh, 2016; Rudenko, 2017; Sudhof, 2018).

Neurexins are a major class of presynaptic cell adhesion molecules (Reissner *et al*, 2013; Sudhof, 2017; Rudenko, 2019), which are genetically linked to multiple psychiatric and neuropsychiatric diseases, including schizophrenia, intellectual disability and Tourette’s syndrome (Schaaf *et al*, 2012; Rees *et al*, 2014; Huang *et al*, 2017; Kasem *et al*, 2018). Neurexins are widely expressed in the nervous system by three genes (*NRXN1‐3*) that each expresses both larger ɑ‐ and shorter β‐neurexins (Ushkaryov *et al*, 1992; Ushkaryov *et al*, 1994; Reissner *et al*, 2013), with *NRXN1* also encoding as a small γ‐neurexin isoform (Yan *et al*, 2015; Sterky *et al*, 2017). α‐Neurexins carry on their extracellular part six LNS (laminin‐NRXN‐sex hormone binding globulin) and three EGF (epidermal growth factor‐like) domains, while β‐neurexins contain only a single LNS domain and Nrxn1γ lacks folded extracellular domains. However, all isoforms encompass a glycosylated “stalk” region containing a conserved cysteine‐loop (Cys‐loop) (Gokce & Sudhof, 2013), followed by a transmembrane domain and a relatively short intracellular sequence ending with a PDZ‐binding motif (Hata *et al*, 1996). Extensive research has identified more than 20 structurally diverse ligands that may participate in neurexin complexes by direct interactions (reviewed in Sudhof, 2017; Rudenko, 2019).

The carbonic anhydrase (CA)‐related protein CA10 constitutes a special type of neurexin ligand by binding robustly and stoichiometrically only when expressed in the same presynaptic neuron (i.e., in *cis*), suggesting that the interaction forms in the secretory pathway. Indeed, a chaperone‐like function is suggested by the finding that overexpressed CA10 could increase neurexin surface levels (Sterky *et al*, 2017). CA10 and its homologue, CA11, are secreted proteins that each contain an enzymatically inactive CA domain (Lovejoy *et al*, 1998; Okamoto *et al*, 2001), similar to the extracellular CA‐like domains of the tyrosine phosphatase receptors R‐PTPγ/PTPRG and R‐PTPζ/PTPRZ1 (Krueger & Saito, 1992; Barnea *et al*, 1993). However, the CA‐like domain of CA10/11 is followed by a unique ~ 25 residues long C‐terminal “tail” that contains a single conserved cysteine. This cysteine can form an intermolecular disulfide between CA10 and the N‐terminal of the two cysteines in the neurexin Cys‐loop (Sterky *et al*, 2017). As CA10 is expressed in subsets of neurons, with highest expression found in cerebellum (Aspatwar *et al*, 2010), it may serve to regulate neurexin complexes in specific cell types. CA10 has been found to be important for normal development in Zebrafish (Aspatwar *et al*, 2015) and has also been shown to suppress glioma growth by an unknown mechanism (Tao *et al*, 2019). However, the exact roles of CA10 during normal development and as part of neurexin complexes remain unknown.

Molecular diversity of neurexin isoforms contributes to functional specialization of specific synapses. For example, alternative splicing at six conserved sites (SS1‐6), generates hundreds—possibly a thousand—of unique neurexin transcripts (Treutlein *et al*, 2014; Schreiner *et al*, 2014). Alternative splicing at a single site (splice site 4; SS4) is sufficient to regulate postsynaptic receptor responses in an isoform‐dependent manner (Aoto *et al*, 2013; Dai *et al*, 2019). Further diversification arises from glycosylation. Recent work has shown that a substantial fraction of neurexins (70–80% in mouse brains) carry a heparan sulfate (HS) glycosaminoglycan (GAG) chain (Zhang *et al*, 2018). The HS‐modified serine is conserved among neurexins and present in ɑ‐, β‐, and γ‐neurexin isoforms, which all can carry this post‐translational modification (Zhang *et al*, 2018; Roppongi *et al*, 2020). The HS chain has been shown to cooperate in the protein–protein interactions between neurexins and postsynaptic neuroligins (Nlgns) and leucine‐rich repeat transmembrane neuronal protein 2 (LRRTM2) to enhance and/or stabilize these interactions (Zhang *et al*, 2018). The HS chain can also recruit additional HS‐binding proteins to neurexin complexes, for example postsynaptic LRRTM4 (Roppongi *et al*, 2020). Whether it may also recruit secreted proteins that influence synapse formation (Yuzaki, 2018), for example, growth factors and signaling molecules known to bind other HSPGs (Esko & Selleck, 2002; Xie & Li, 2019), remains unknown. While more remains to be learned about this modification, it is clearly important for at least some of the synaptic functions of neurexins. For example, mice that lack HS on Nrxn1 show structural and functional impairments of hippocampal mossy fiber‐CA3 synapses (Zhang *et al*, 2018).

Biosynthesis of HS begins with the conjugation of a xylose residue to a serine on the core protein by *O‐*xylosyltransferase activity, accounted for in vertebrates by one of two xylosyltransferases (XYLT1/2) (Esko & Selleck, 2002; Briggs & Hohenester, 2018). Additional glycosyltransferases in turn attach two galactose and one glucuronic acid sugars to form the tetrasaccharide core structure that is shared between all GAGs and is the starting point for further chain elongation (Kreuger & Kjellén, 2012). In this process, alternating glucuronic acid (GlcA) and *N*‐acetylglucosamine (GlcNAc) residues are added, resulting in a 40–100 residues linear polysaccharide chain. Different enzymatic modifications, including epimerization, deacetylation and sulfation give rise to the mature chain and functionally specialized segments. For example, highly sulfated regions preferentially engage with specific interacting proteins (Xu & Esko, 2014). How the neurexin HS chain may be modified and how this relates to its function at the synapse is not known. Also unknown is whether the neurexin HS chain is ubiquitous to all neurexins or regulated to be expressed only in select cell types or at specific synapses.

In this work, we observe that the addition of HS to neurexin can be regulated by CA10. We find that CA10 can block the neurexin HS addition by directly binding to neurexin before HS *en route* in the secretory pathway. Resulting non‐HS neurexin showed reduced binding to LRRTM2 and capacity for Nlgn1‐mediated synapse formation, demonstrating that CA10 can modify synaptic properties of neurexins. Localized protein–protein interactions within the secretory pathway, such as that between CA10 and neurexin, exemplifies a cell‐biological mechanism able to directly control substrate‐specific glycosylation without affecting global proteoglycan biosynthesis.

## Results

### Neurexin HS addition depends on residues within its Cys‐loop

**Figure 1:**
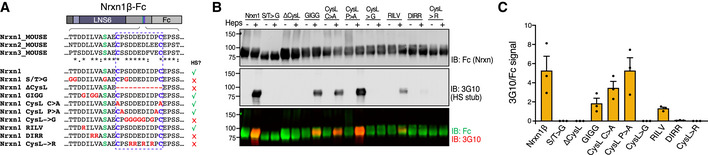
Analysis of Nrxn1 sequence determinants required for HS addition Sequences of secreted Fc‐tagged Nrxn1β variants analyzed. Mutated residues are shown in red, the HS‐modified serine in green, and cysteines forming the Cys‐loop in blue. Right column (‘HS?’) summarizes the results from (B, C). Asterisks, colons, and periods indicate fully, strongly, or weakly conserved residues, respectively.Representative immunoblot of Nrxn1β‐Fc variants harvested from HEK293 cell media and subjected to heparinase treatment (‘Heps’). Samples were analyzed by reducing SDS–PAGE and immunoblotting with antibodies against the Fc tag and the 3G10 epitope (to detect the HS stub which reveal HS after heparinase digestion).Quantification of 3G10 (reflecting HS) signal normalized to that of Fc (Nrxn1). Bar graph shows means ± SEM of 3 independent experiments.

**Figure EV1:**
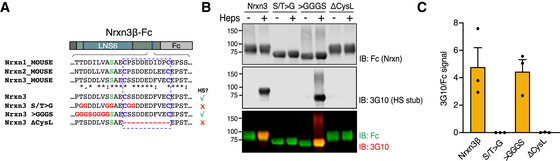
Analysis of Nrxn3 sequences required for its HS addition (related to Fig 1) Sequences of secreted Fc‐tagged Nrxn3β variants used. Mutated residues are shown in red, the HS‐modified serine in green, and cysteines forming the Cys‐loop in blue. Asterisks, colons, and periods indicate fully, strongly, or weakly conserved residues, respectively. Right column (‘HS?’) summarizes the results from (B, C).Representative immunoblot of Nrxn3β‐Fc variants harvested from HEK293 cell media and subjected to heparinase treatment (“Heps”). Samples were analyzed by reducing SDS–PAGE and immunoblotting with antibodies against the Fc tag (Nrxn) and the 3G10 epitope (for the HS stub).Quantification of 3G10 (reflecting HS) signal normalized to that of Fc (Nrxn). Bar graph shows means ± SEM of 3 independent experiments.

**Figure EV2:**
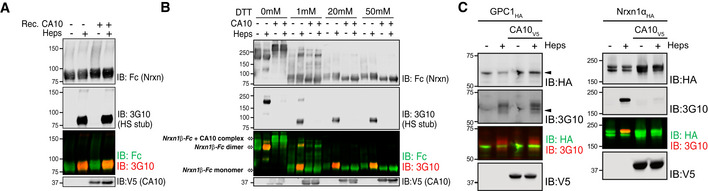
Heparinase digestion and detection of the HS stub is not inhibited by CA10 and prevention of HS addition by CA10 is specific for neurexins (related to Fig 2) Fc‐tagged Nrxn1β was captured on protein A beads and digested with heparinases (“Heps”) with or without recombinant V5‐tagged CA10 (3.5 μM final concentration). Samples were analyzed by SDS–PAGE under reducing conditions, followed by immunoblotting against the Fc tag and the HS stub (3G10).Fc‐tagged Nrxn1β expressed in HEK293 cells, alone or together with CA10‐V5, were captured on protein A beads and treated with heparinases (“Heps”) in the presence of indicated concentrations of DTT (to dissociate the Nrxn1β‐CA10 complex). Samples were analyzed by SDS–PAGE under non‐reducing conditions, followed by immunoblotting against the Fc tag and the HS stub (3G10). DTT at a concentration of 20 mM fully dissociated the CA10‐ Nrxn1β complex without inhibiting heparinase activities.HA‐tagged GPC1 (left) or HA‐Nrxn1α, processed in parallel as a control (right), were expressed alone or together with V5‐tagged CA10 in HEK293 cells, immunoprecipitated for HA and subjected to heparinase treatment (“Heps”) to reveal HS‐carrying isoforms (similar to the experiment outlined in Fig 2A). Samples were analyzed by immunoblotting with antibodies against HA, the 3G10 epitope (for the HS stub) and V5. Non‐HS GPC1 is marked by an arrowhead, while HS‐containing GPC1 is not visible prior to heparinase treatment (Wen *et al*, 2014).

**Figure EV3:**
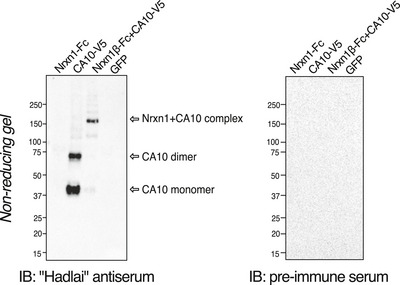
Characterization of the Nrxn1‐CA10 antiserum (related to Fig 3) Soluble Nrxn1γ ‐Fc, CA10‐V5 and both were expressed in HEK293 cells. Media was analyzed by non‐reducing SDS–PAGE and blotted with “Hadlai” antiserum raised against recombinant covalent Nrxn1γ‐CA10 complex or pre‐immune serum from the same rabbit. The antiserum recognizes CA10 and the Nrxn1‐CA10 complex, but not Nrxn1γ alone.

**Figure EV4:**
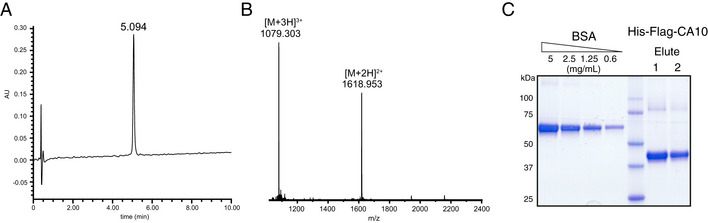
Analytical characterization of recombinant CA10 and the Nrxn1 stalk peptide (related to Fig 4) Analytical HPLC chromatogram of the purified Nrxn1 stalk peptide.ESI mass spectrum of the purified Nrxn1 stalk peptide. The calculated average mass is 3,237.35 [M + H]^+^.Coomassie‐stained gel of recombinant HIS‐FLAG‐tagged CA10, separated under reducing conditions.

**Figure EV5:**
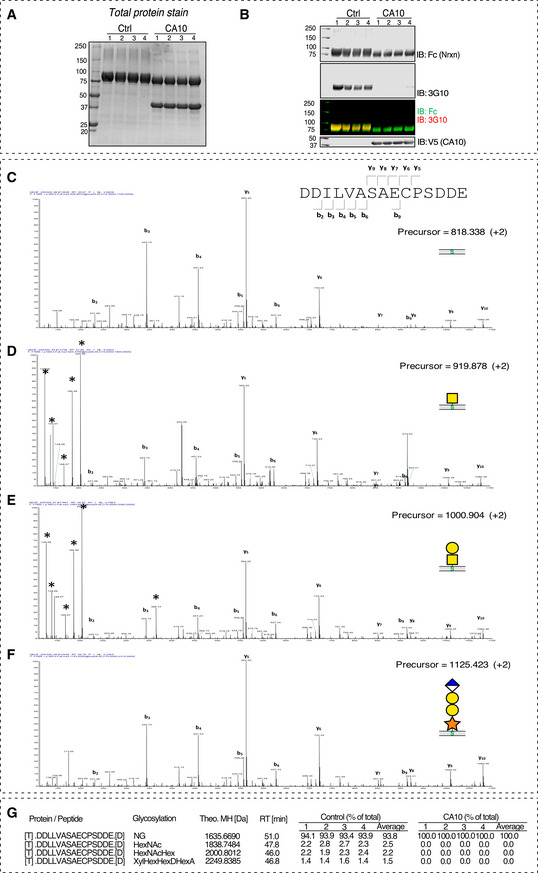
MS/MS spectra of peptides from recombinant Nrxn1 expressed with or without CA10 (related to Fig 5) A, BSDS–PAGE gel stained for (A) total protein and (B) immunoblot of the replicate experiments used for MS/MS analysis.CFragment spectrum of the unmodified DDILVASAECPSDDE peptide, precursor at *m*/*z* 818.338 ([M + 2H]^2+^).DFragment spectrum of the DDILVASAECPSDDE+ HexNAc, precursor at *m*/*z* 919.878 ([M + 2H]^2+^).EFragment spectrum of the DDILVASAECPSDDE+ HexNAcHex, precursor at *m*/*z* 1,000.904 ([M + 2H]^2+^).FFragment spectrum of the DDILVASAECPSDDE+ XylGalGalGlcA‐H_2_O, precursor at *m*/*z* 1,125.423 ([M + 2H]^2+^).GTable with theoretical MH and retention times (RT) of each glycopeptide, and % total occupancy of each replicate in the control and CA10 samples (values represented in Fig 5D). Data information: All fragment spectra (C–F) display high similarity with the same major peptide fragments being observed, confirming the peptide identities. The *O*‐glycosylated peptides further displayed oxonium ions associated with mucin‐type *O*‐GalNAc glycosylation.

To learn more about neurexin and its heparan sulfate (HS), we analyzed the sequence context required for this post‐translational modification. We used a set of secreted, Fc‐tagged neurexin‐1β variants with different mutations in residues surrounding the HS‐modified serine residue (Fig 1A). The proteins were expressed in HEK293 cells and purified from the media using protein A beads. The samples were subjected to on‐bead digestion with heparinases and analyzed by immunoblotting under reducing conditions. Heparinase treatment did not result in detectable size shifts, consistent with the previous observations that only a minor fraction of neurexin expressed in HEK293 cells contain HS (Zhang *et al*, 2018). Instead, we relied on a monoclonal antibody which detects the HS “stub” that remains after heparinase digestion [3G10 epitope; (David *et al*, 1992)] and that allowed us to detect species that carry HS against the background of non‐HS species (Fig 1B). Using this assay, we found that wild‐type neurexin‐1 contained HS as expected, but not a negative control in which the modified serine was mutated (Fig 1B and C). The serine is located just N‐terminal of a Cys‐loop (Gokce & Sudhof, 2013) that is predicted to form between two conserved cysteines flanking a stretch of negatively charged residues. Deletion of the entire Cys‐loop (ΔCysL) or the acidic residues within it (CysL>G, CysL>R) blocked HS addition. However, mutating both cysteines (CysL C>A) to prevent the loop to form had no effect on HS addition, suggesting a requirement for residues within the loop rather than the loop itself. Moreover, we found that the leucine–valine residues (DILV) N‐terminal of the HS‐modified serine could be mutated to glycines (GIGG), but not to positively charged arginines (DIRR) (Fig 1B and C). We observed a similar tolerance for glycines N‐terminal of the serine when testing variants of neurexin‐3β (Fig EV1, EV2, EV3, EV4, EV5).

The conserved leucine–valine residues in positions −3 and −2 relative to the HS‐modified serine, as well as the cysteine in position +3, are both required for the binding of CA10 to neurexins (Sterky *et al*, 2017). This observation, together with the finding that exogenous CA10 expression dramatically shifts neurexin isoform distribution in neurons (Sterky *et al*, 2017), prompted us to investigate whether CA10 may play a role in regulating the addition of HS to neurexin.

### CA10 blocks addition of HS to neurexin

**Figure 2:**
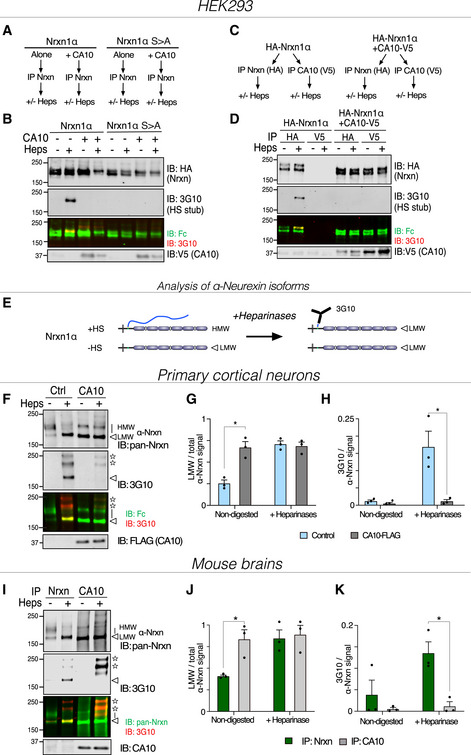
CA10 prevents HS addition to neurexin A–EHEK293 cells: (A) Schematic outline of the experiment shown in (B). HA‐Nrxn1α or HA‐Nrxn1α (S > A), a mutant of the modified serine were expressed alone or in combination with V5‐tagged CA10 in HEK293 cells. Neurexins were immunoprecipitated for HA and treated with heparinases (‘Heps’). (B) Representative immunoblots (of three independent experiments) with antibodies against HA, the 3G10 epitope (for the HS stub) and V5. (C) Schematic outline of the experiment shown in (D). HA‐Nrxn1α was expressed alone or in combination with CA10‐V5 in HEK293 cells, lysates were immunoprecipitated for HA (for Nrxn1α) or V5 (for CA10). (D) Representative immunoblots (of three independent experiments) analyzed as described for (B). (E) Schematic illustration of how endogenous neurexin HS was analyzed. HS‐modified α‐neurexin migrates at higher apparent molecular weight (HMW) than non‐modified low molecular weight (LMW; white arrowheads) species (left). Following treatment with heparinases (right), both HS‐modified and non‐modified α‐neurexin migrates as LMW species, but HS‐modified α‐neurexin can be detected by the 3G10 monoclonal.F–HPrimary cortical neurons: Cortical neurons were infected with control lentivirus (empty vector) or lentivirus expressing FLAG‐tagged CA10, and cell lysates subjected to immunoprecipitation for neurexin and heparinase treatment. (F) Samples were analyzed by immunoblotting using antibodies against neurexins, 3G10 (for the HS stub) and FLAG (for CA10). Stars indicate non‐neurexin bands (see Results). (G) Quantification of low molecular weight neurexin (LMW; white arrowhead) in relation to total neurexin amounts [upper band (line) + LMW]. Data shown as mean ± SEM (*n* = 3 independent experiments); *P < 0.05 by 2‐way ANOVA and Holm–Sidak tests. (H) Quantification of the 3G10 signal normalized to the signal of the corresponding neurexin band. Data shown as mean ± SEM of biological replicates. *P < 0.05 by 2‐way ANOVA and Holm–Sidak tests (*n* = 3).I–KMouse brains: Neurexins and CA10 were immunoprecipitated from total mouse brain lysates and subject to heparinase treatment. (I) Samples were analyzed by immunoblotting using antibodies against neurexins, the 3G10 epitope (for the HS stub) and CA10. Stars indicate non‐neurexin bands (see Results). (J) Quantification of low molecular weight neurexin (LMW; white arrowhead) in relation to total neurexin amounts [upper band (line) + LMW]. Data shown as mean ± SEM (*n* = 3 independent experiments); *P < 0.05 by 2‐way ANOVA and Holm–Sidak tests. (K) Quantification of the 3G10 signal normalized to the signal of the corresponding neurexin band. Data shown as mean ± SEM of 3 biological replicates. *P < 0.05 by 2‐way ANOVA and Holm–Sidak tests.

To assess whether CA10 had an effect on neurexin HS, we expressed hemagglutinin (HA)‐tagged Nrxn1α alone or in combination with V5‐tagged CA10 in HEK293 cells. Deletion of the modified serine (S > A) was used as a negative control. We then immunoprecipitated neurexin from cell lysates and detected species carrying HS using the monoclonal antibody 3G10, after treatment with heparinases (Fig 2A). We readily detected HS‐carrying neurexin in cells that expressed wildtype Nrxn1α alone, but not the (S > A) mutant (Fig 2B). However, no HS‐carrying neurexin could be detected in cells that also expressed CA10, suggesting that CA10 completely blocked the formation of HS‐carrying species. To further corroborate that CA10‐bound neurexin did not carry HS, we immunoprecipitated CA10 by means of its V5‐tag and analyzed the co‐immunoprecipitated Nrxn1α (Fig 2C). As expected, no HS‐carrying Nrxn1α was detected in the complex (Fig 2D).

Because the abovementioned experiments rely on trimming of HS by heparinases to reveal the neo‐epitope recognized by the 3G10 monoclonal, we performed several control experiments to exclude that CA10 may inhibit heparinase activity. First, we expressed secreted Nrxn1β in HEK293 cells, as described above, and added recombinant CA10 prior to the heparinase digestion step. Supplemented CA10 had no noticeable effect on the detection of HS‐ carrying species (Fig EV2A). As CA10 and neurexins can form an intermolecular disulfide bond when co‐expressed (Sterky *et al*, 2017), we also considered the possibility that covalently bound CA10 may prevent access to the HS chain by heparinases. To test this, we co‐expressed Fc‐tagged Nrxn1β and V5‐tagged CA10, then partially dissociated the complexes with increasing amounts of DTT, treated samples with heparinases and analyzed by SDS–PAGE under non‐reducing conditions. The Nrxn1β‐CA10 complexes were completely dissociated by 20 mM DTT. At the same time, concentrations of up 50 mM had no noticeable effect on heparinase activities, as the 3G10 epitope could be detected when neurexin was expressed alone (Fig EV2B). No HS could be detected on neurexin dissociated from complexes with CA10. Thus, CA10 blocks the biosynthesis of the HS chain on neurexins. Moreover, we tested whether CA10 also blocks biosynthesis of another HSPG, glypican‐1 (GPC1). We expressed HA‐tagged GPC1 alone or together with CA10 in HEK293 cells and analyzed the HS of GPC1 as described for Nrxn1α (Fig 2A). In contrast to Nrxn1α, CA10 did not affect the levels of GPC1 that carry HS (Fig EV2C).

To test whether CA10 could block HS added on endogenous neurexin, we studied mixed neuron/glia cultures from mouse cortex. Cells were harvested after 14 days *in vitro* and neurexin was immunoprecipitated from the lysates, heparinase‐treated and detected using a pan‐neurexin antibody. Endogenous α‐neurexins, which dominate in mouse brains (Anderson *et al*, 2015; Sterky *et al*, 2017), migrate on SDS–PAGE gels as both a distinct lower band and more diffuse bands of ~ 30 kDa higher apparent molecular mass (Zhang *et al*, 2018). Digestion of the HS GAG chain with heparinases compressed most (but not all) of the apparently larger isoforms to the lower band (open arrowheads; Fig 2E and F). The relative amounts of the lower isoforms increased from ~ 25 to ~ 70%, indicating that ~ 50% of α‐neurexins in our cultures carry the HS chain (Fig 2F). Exogenous expression of FLAG‐tagged CA10 by lentiviral transduction resulted, as shown previously (Sterky *et al*, 2017), in a redistribution of α‐neurexin isoforms that mimicked treatment with heparinases. In this case, the distribution of isoforms did not shift further upon heparinase treatment (Fig 2F and G). Moreover, the 3G10 monoclonal showed no reactivity toward neurexin from CA10‐expressing cells, indicating that CA10 fully blocked formation of HS‐carrying neurexin in neurons (Fig 2F and H). However, consistent with what has been observed by others (Zhang *et al*, 2018), two 3G10‐reactive bands that did not correspond to neurexin isoforms consistently appeared following immunoprecipitation and heparinase treatment, possibly reflecting a background of other abundant HSPGs.

The above results suggest that CA10 may regulate neurexin‐HS formation. If so, endogenous CA10 would exclusively associate with non‐HS neurexin. To test this, we subjected mouse brain lysates to immunoprecipitations using either a pan‐neurexin antibody or an antiserum raised against the CA10‐Nrxn1γ complex (Fig EV3). The resulting fractions, representing the total pool of neurexin as well as neurexin bound to CA10, were subject to heparinase treatment and analyzed by immunoblotting (Fig 2I). As expected, the pan‐neurexin antibody immunoprecipitated both HS‐carrying and non‐HS neurexin isoforms. In contrast, CA10 exclusively immunoprecipitated neurexin of lower molecular mass that did not shift in size upon heparinase treatment (Fig 2I and J). Furthermore, no 3G10‐reactive α‐neurexin could be detected in fractions immunoprecipitated for CA10 (Fig 2I and K). Thus, CA10 exclusively binds non‐HS neurexins *in vivo*.

### A direct non‐covalent interaction is necessary and sufficient for CA10 to prevent neurexin HS

**Figure 3:**
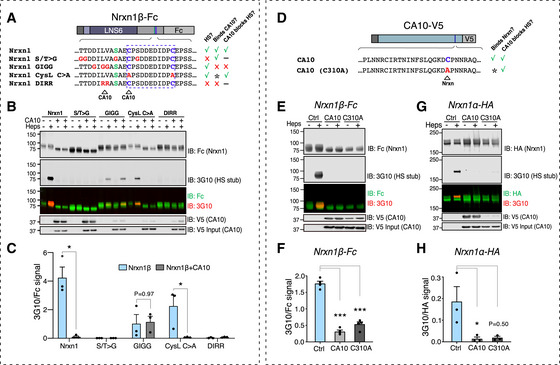
A direct CA10‐neurexin interaction is required to prevent neurexin HS addition, and a non‐covalent interaction is sufficient ASequences of the Nrxn1β mutants used, color‐coded as in Fig 1A, with residues previously mapped to bind CA10 indicated. Right, table summarizing properties of the recombinant variants: ‘HS?’ indicates if HS‐modified when expressed alone (Fig 1B), ‘Binds CA10?’, indicates if found to bind CA10, according to (Sterky *et al*, 2017), and whether the HS modification is blocked by CA10 (this figure). Asterisk denotes non‐covalent interaction (see discussion).BRepresentative immunoblot of the indicated proteins harvested from HEK293 cell media and subjected to heparinase treatment (‘Heps’). Samples were analyzed by reducing SDS–PAGE and immunoblotting with antibodies against Fc (for Nrxn1β), the 3G10 epitope (for the HS stub) and V5 (for CA10). Input samples were analyzed separately for CA10 to ensure equal expression (lower panel).CQuantification of the 3G10 epitope signal, normalized to Fc (Nrxn1β). Data shown are mean ± SEM of 3 independent replicates. *P < 0.05; by Mann–Whitney tests comparing each Nrxn1 mutant with and without CA10 (*n* = 3). No significance difference was observed for S&T > G (*P* = 0.1143), GIGG (*P* = 0.9714), or DIRR (*P* = 0.4857) variants.D–HFc‐tagged (secreted) Nrxn1β and HA‐tagged (transmembrane) Nrxn1α were co‐expressed with CA10 and a CA10 mutant for the reactive cysteine CA10(C310A). (D) Summary of CA10 variants used, and their properties (this figure). Asterisk denotes non‐covalent interaction (see Discussion). Panels (E) and (G) show representative immunoblots analyzed using antibodies against the Fc tag (for Nrxn1β‐Fc) or the HA tag (Nrxn1α‐HA), respectively, the 3G10 epitope (for the HS stub) and V5 (CA10‐V5), as indicated. CA10 in input samples is shown for demonstration of equal expression. (F, H) Quantification of the 3G10 epitope signal normalized to Fc (for Nrxn1β‐Fc) or HA (for Nrxn1α‐HA) signals, respectively. Data shown are mean ± SEM of 3 biological replicates. *P < 0.05; ***P < 0.001 by 2‐way ANOVA and Holm–Sidak tests.

To further study how CA10 prevents neurexin HS, we used a set of soluble Fc‐tagged Nrxn1β (Fig 3A) that were co‐expressed with V5‐tagged CA10, and analyzed as previously described. Co‐expression with CA10 fully blocked HS addition to wildtype neurexin, consistent with previous results. In contrast, the GIGG neurexin mutant, to which CA10 does not bind (Sterky *et al*, 2017), became HS‐carrying also in presence of CA10 (Fig 3B and C). This result suggests that CA10 exerts its effect by means of a direct interaction with neurexin. CA10 could, however, block HS addition to a neurexin Cys‐loop (CysL C>A) mutant. This surprised us, as one of the cysteines in the Cys‐loop participates in the formation of the intermolecular disulfide with CA10 and the same mutant failed to robustly bind to CA10 in previous experiments (Sterky *et al*, 2017), as well as current immunoprecipitations (Fig 3B; lower panels). We hypothesized that a non‐covalent interaction between CA10 and neurexin, which is believed to precede formation of the covalent bond, is sufficient to prevent HS addition. To explore this possibility, we mutated the neurexin‐binding cysteine in the C‐terminal tail of CA10 (C310A; Fig 3D). First, we tested this mutant in the assay using secreted Fc‐tagged Nrxn1β and found that it could block the addition of HS to wild‐type Fc‐tagged Nrxn1β equally well as wild‐type CA10 (Fig 3E and F). We additionally tested the CA10(C310A) mutant in HEK293 cells expressing HA‐tagged (transmembrane) Nrxn1α. Also here, we found that it prevented the addition of HS almost as efficiently as wild‐type CA10, despite significantly less of it co‐immunoprecipitated with neurexin (Fig 3G and H). From this, we conclude that a direct CA10–neurexin interaction, presumably in the early secretory pathway, is required, but covalent binding is not.

### Analysis of the Nrxn1 stalk and its interaction with CA10 by NMR

**Figure 4:**
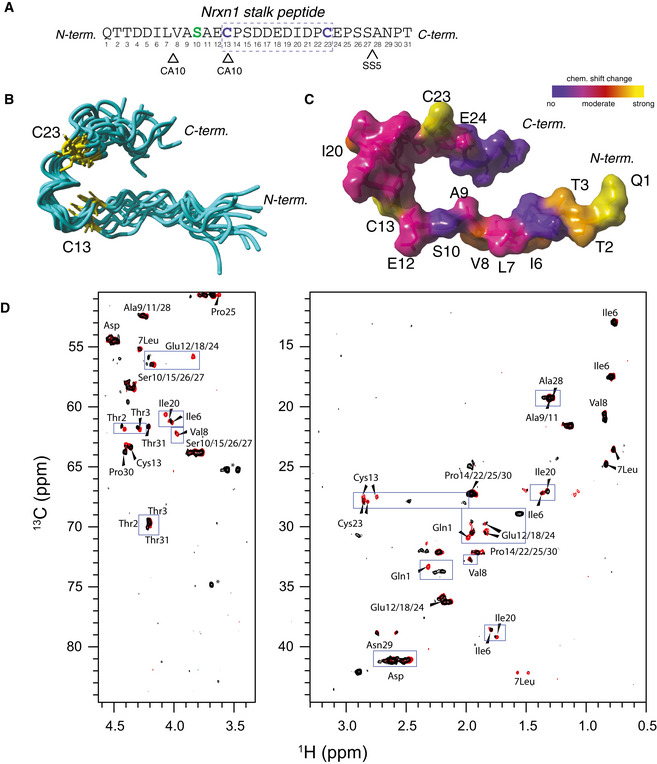
Analysis of the Nrxn1 stalk and the neurexin–CA10 interaction by NMR Sequence of the Nrxn1 peptide used for the analysis. Residues previously mapped to bind CA10 (Sterky *et al*, 2017) and the site where alternative splice site 5 (SS5; Ushkaryov *et al*, 1992) inserts three residues (GGL) are indicated.Solution structure of the Nrxn1 stalk, with cysteines labeled yellow. The 10 models of lowest energy with respect to the target function are shown (ensemble average RMSD 2.95 Å).Chemical shift changes induced by CA10, qualitative mapped onto the Nrxn1 solution structure. Residues with the strongest changes are labeled.Comparison of ^1^H‐^13^C HSQC spectra of Nrxn1 stalk (red) and Nrxn1 stalk/CA10 (black) at a 2:1 molar ratio (50 mM phosphate buffer, pH 7.4, 0.5 mM d10‐DTT, 10% D_2_O). Nrxn1 residues with stronger chemical shift changes are indicated blue boxes. Asterisk (*) indicates an impurity from glycerol.

To begin to understand more about the CA10–neurexin interaction, we employed NMR. We generated a synthetic peptide corresponding to the part of the Nrxn1 stalk region that contains the Cys‐loop, the CA10 binding site, and the HS‐modified serine (Figs 4A, and EV4CA and B) and determined its structure in water. All proton resonances could be assigned except for those on the four proline residues, which had almost identical chemical shifts (Table EV2). As expected, most ^13^C chemical shifts indicate a random coil and the structure calculation resulted in a rather undefined structural ensemble in solution (RMSD 2.9 Å, Fig 4B). However, residues within the predicted Cys‐loop (residues 13 to 23) seemed to adapt a defined loop‐like structure (residues 12–24 RMSD 1.8 Å). The peptide was kept in a reduced state during the experiment, but the structure puts the two cysteines in relative proximity and it can easily be envisioned how the loop may adopt a “closed” confirmation by disulfide formation under permissive conditions.

Next, we studied which residues in the Nrxn1 stalk peptide are primarily affected by binding to CA10. We purified recombinant CA10 from HEK293 cells (Fig EV4C), mixed the protein with the Nrxn1 peptide and analyzed changes in the NMR spectra (Fig 4C and D). Measurements were conducted under reducing conditions to reduce complexity that may be induced by intra‐ and intermolecular disulfide bond formation. CA10 induced strong chemical shift changes at the N terminus of the peptide as well as the residues previously shown to be important for its binding: the leucine–valine residues and the Cys‐loop (Fig 4C and D). Within the Cys‐loop, the cysteines themselves displayed the strongest shift changes, together with the iso‐leucine. The chemical shift of the HS‐modified serine appeared unaltered. These results support the notion that CA10 engages residues both N‐ and C‐terminal of the HS‐modified serine, while not binding to the serine itself.

### CA10 prevents HS biosynthesis by blocking neurexin xylosylation

**Figure 5:**
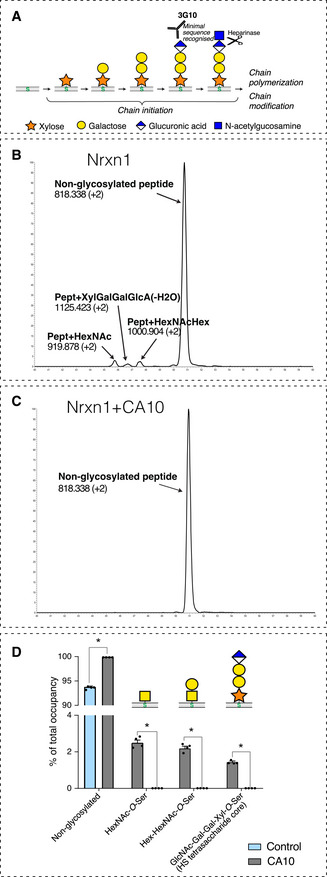
CA10 prevents neurexin xylosylation ASchematic representation of the intermediate steps of HS biosynthesis. Heparinase cleavage and the neo‐epitope detected by the 3G10 antibody following cleavage (David *et al*, 1992) are indicated.B, CExtracted ion chromatograms (EIC) of DDILVASAECPSDDE peptide and its glycosylated forms, from Nrxn1β expressed in HEK293 cells. Fragment ion spectra for all observed glycosylated peptides are shown in Fig EV5.DQuantification, expressed as % of total occupancy of each glycosylation form, upon control and CA10 expression conditions. Bar graph shows means ± SEM of 4 biological replicates; *P < 0.05 by Mann–Whitney tests comparing the occupancy of each glycosylation with and without CA10 (*n* = 4 biological replicates).

The above experiments suggest that CA10 can block formation of the neurexin HS chain by a direct interaction, but does not demonstrate at what step HS biosynthesis is blocked. The minimal epitope that can be recognized by the 3G10 monoclonal antibody is a tetrasaccharide structure, so assays that rely on it will be unable to distinguish which step leading up to this structure that may be blocked (Fig 5A). We therefore used mass spectrometry (MS) to directly determine which exact saccharide residues (if any) that were added to the target serine in the presence of CA10. We expressed Fc‐tagged Nrxn1β in HEK293 cells alone or in combination with CA10 (Fig EV5A and B), pulled down on protein A beads, followed by digestion with Asp‐N and a combination of heparinases (for increased processivity). The resulting peptides were analyzed by label‐free, quantitative LC‐MS/MS. We searched for peptides with *m/z* values corresponding to possible glycosylations (Fig 5A). We consistently detected a species corresponding to the full GlcNAc‐Gal‐Gal‐Xyl‐*O*‐Ser HS tetrasaccharide core structure (Figs 5B and EV5F). However, we also detected HexNAc‐*O*‐Ser and Hex‐HexNAc‐*O*‐Ser species, presumably corresponding to GalNAc‐ and Gal/GalNAc‐modified species (Figs 5B, and EV5D and E). Thus, HEK293 cells alternatively subject the same serine to mucin‐type GalNAc glycosylation, which may reflect the cell type’s inefficient biosynthesis of HS on to exogenous neurexin. However, most detected peptides were non‐glycosylated (Figs 5B and EV5C). In the presence of CA10, no glycosylated species were detected (Figs 5C and D, and EV5G). From this, we conclude that CA10 prevents the primary step of HS biosynthesis: the xylosylation of the serine residue. Viewed together, this suggests that CA10 form a complex with neurexin *en route* early in the ER to prevent xylosylation of the serine in the late ER and Golgi by steric hindrance.

### Neurexin HS is differentially regulated across brain regions

**Figure 6:**
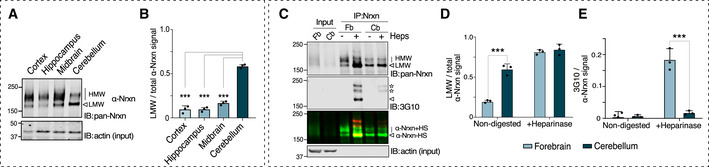
Neurexin HS modification is differentially regulated between brain regions Immunoblot α‐showing neurexin isoform distribution in cortex, hippocampus, midbrain, and cerebellum dissected from 8‐week‐old mice.Quantification of lower molecular weight neurexin (LMW; white arrowhead) in relation to total levels of α‐neurexins [upper band (line) + LMW]. Bar graphs show mean ± SEM of 3 independent replicates ***P < 0.001 by 2‐way ANOVA and Holm–Sidak tests, comparing to levels in cerebellum.α‐Neurexin immunoprecipitated from forebrain (Fb) and cerebellum (Cb) using an anti‐pan‐neurexin antibody was heparinase‐treated (“Heps”) and analyzed by immunoblotting with antibodies against neurexin and the 3G10 monoclonal (to detect the HS stub). Stars indicate non‐neurexin bands (see Results).Quantification of low molecular weight neurexin (LMW; white arrowhead) in relation to total levels of α‐neurexins [upper band (line) + LMW]. Bar graphs show mean ± SEM of 3 biological replicates. ***P < 0.001 by 2‐way ANOVA and Holm–Sidak tests comparing forebrain and cerebellum.Quantification of the 3G10 signal normalized to the signal of the corresponding neurexin band. Bar graphs show mean ± SEM of 3 biological replicates. ***P < 0.001 by 2‐way ANOVA and Holm–Sidak tests comparing forebrain and cerebellum.

The finding that CA10 blocks neurexin xylosylation and HS addition suggests that CA10 is not simply a chaperone‐like protein for neurexins but rather serve to regulate the relative levels of neurexin molecules that carry the HS chain, possibly in a cell type‐ and brain region‐specific manner. The relative levels of HS neurexin have previously been suggested to be stable throughout mouse postnatal development (Zhang *et al*, 2018). However, it is not known whether the relative levels differ between specific cell types and synapses. As an initial assessment of this, we dissected mouse brain regions and analyzed the isoform distribution of α‐neurexins (Fig 6A). We found that a fraction migrated at as a sharp band species of lower molecular mass, indicative of being non‐glycosylated. Levels of this seemingly non‐glycosylated α‐neurexins were low in cortex and hippocampus (~ 10%), slightly higher in midbrain (~ 20%), but significantly higher in the cerebellum (~ 60%) (Fig 6B). To confirm that the size‐shift indeed depended on the HS modification, we immunoprecipitated neurexins from cerebellum as well as the other brain regions combined (collectively referred to as the forebrain), and digested the samples with heparinases (Fig 6C). As expected, heparinase treatment redistributed, from higher to lower molecular mass, isoforms in forebrain samples, but surprisingly no corresponding shift was seen in cerebellar samples (Fig 6C and D). Furthermore, analysis by immunoblotting for the 3G10 epitope did not reveal almost any HS‐modified neurexin in cerebellar samples, although readily detected in forebrain samples (Fig 6C and E). Thus, the neurexin HS modification is different in different brain regions. Interestingly, CA10 has its highest levels in cerebellum (Aspatwar *et al*, 2010; Sterky *et al*, 2017), likely reflecting the low levels of HS‐modified neurexin we observed there.

### Role of CA10 in regulating neurexin–ligand interactions

**Figure 7:**
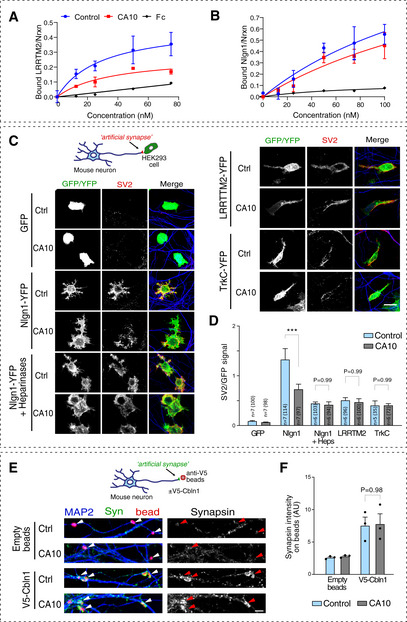
CA10 reduces affinity to postsynaptic partners LRRTM2 and neuroligin‐1 and attenuates neurexin‐induced synapse formation A, BCell surface binding to endogenous neurexins. (A) Recombinant Fc‐tagged LRRTM2 was added to day 10 hippocampal neurons expressing CA10 or control (empty vector) by lentiviral transduction. Surface‐bound protein was quantified as the Fc signal of and normalized to a staining for endogenous neurexin (*n* = 4 cultures; three fields imaged and averaged per well). Scatchard analysis for LRRTM2 revealed an apparent Kd of 29.5 nM and a B_max_ of 0.49 nM for control and Kd 35.3 nM, B_max_ 0.28 nM upon expression of CA10; significant (*P* = 0.0006) by non‐linear regression. Data shown as mean ± SEM of biological replicates (*n* = 4). (B) Increasing amounts of recombinant, Fc‐tagged Nlgn1 was added to immature hippocampal neurons expressing CA10 or control (empty vector) by lentiviral transduction, and analyzed as in (A). The apparent Kd for Nlgn1 was 186 nM and B_max_ 1.65 nM for control and Kd 223 nM, B_max_ 1.49 nM when CA10 was overexpressed. The difference was not significant (*P* = 0.20) by non‐linear regression. Data shown as mean ± SEM of biological replicates (*n* = 4).C, DArtificial synapse formation assays. (C) Confocal images of hippocampal neurons transduced to express CA10‐FLAG or control (empty vector) and co‐cultured with HEK293 cells expressing GFP, Nlgn1‐YFP, LRRTM2‐YFP or TrkC‐YFP. Cells were stained with antibodies against the synaptic marker SV2 (red) and somatodendritic MAP2 (blue); GFP/YFP is shown in green. Scale bar 20 μm. (D) Quantifications of SV2 signal overlapping GFP/YFP‐positive cells. Data shown as means ± SEM with the *n*, reflecting number of independent cover slips analyzed, indicated in each bar with the total number of cells imaged in parenthesis. Comparisons were made by one‐way ANOVA with Holm–Sidak’s *post hoc* test for multiple comparisons between the indicated groups (GFP condition was omitted from analysis due to unequal variance; ***P < 0.001.E, FAnalysis of cerebellin(Cbln)‐induced artificial synapse formation. (E) Confocal images of hippocampal neurons transduced to express CA10‐FLAG or control (empty vector) and co‐cultured exposed to anti‐V5‐coupled beads pre‐incubated with recombinant V5‐tagged Cbln1 or empty anti‐V5 beads. Cells were stained with antibodies against the synaptic marker synapsin (green), somatodendritic MAP2 (blue); beads are colored red and indicated by arrowheads. Scale bar 10 μm. (F) Quantifications of the synapsin signal overlapping red beads. Data shown as means ± SEM (*n* = 3 independent experiments, each data point corresponding to 58–171 analyzed beads), with comparisons made by two‐way ANOVA with Holm–Sidak’s *post hoc* test for multiple comparisons between the indicated groups.

Does the opposite relation between CA10 and HS‐carrying neurexin affect synaptic functions of neurexins? To test this, we studied how CA10 affects some of the known ligand interactions of neurexin. Both Nlgns and LRRTMs have previously been reported to engage with the neurexin HS via distinct HS‐binding sites (Zhang *et al*, 2018; Roppongi *et al*, 2020). We thus assessed binding of recombinant LRRTM2 and Nlgn1 to endogenous neurexin on the surface of immature hippocampal neurons. Hippocampal neurons transduced with CA10‐expressing or control lentiviruses were exposed to increasing amounts of Fc‐tagged LRRTM2 or Nlgn1. After 1 h of incubation, cells were fixed and labeled for the Fc tag and neurexin, used for normalization. We found that CA10 significantly reduced surface binding of LRRTM2 to endogenous neurexins (Fig 7A). Binding to Nlgn1 showed a similar trend, but was not significant (Fig 7B). Levels of immuno‐detected total neurexin levels were similar between conditions (Appendix Fig S1), indicating that altered affinities accounted for the differences.

Next, we analyzed how CA10 affected the ability of neurons to form neurexin‐induced hemi‐synapses (“artificial synapses”), which readily formed onto non‐neuronal cells that express postsynaptic ligands to neurexins (Biederer & Scheiffele, 2007). We generated primary mouse hippocampal cultures that expressed FLAG‐tagged CA10 or empty control by lentiviral transduction. At DIV10, HEK293 cells transfected to express either neuroligin‐1, LRRTM2, NT‐3 growth factor receptor (TrkC), or GFP were co‐cultured with the neurons for one additional day, followed by fixation and immunolabeling of synapses using an antibody against the synaptic vesicle glycoprotein 2A (SV2A) (Fig 7C). As expected, LRRTM2, Nlgn1 and TrkC all robustly induced the assembly of presynaptic specializations onto contacting axons. CA10 reduced synapse formation induced by Nlgn1 (Fig 7D). Exposure to recombinant heparinases during the co‐culture period attenuated this difference, suggesting that the effect of CA10 is mediated by its ability to block formation of HS on neurexins (Fig 7D). Synapse formation induced by TrkC, which does not rely on neurexins but instead induce synapses by binding to the presynaptic adhesion receptor R‐PTPσ/PTPRS (Takahashi *et al*, 2011), was unaffected. In this experiment, CA10 had no effect on the formation of synapses induced by LRRTM2, perhaps as the protein–protein interaction between neurexins (lacking the SS4 splice insert) and LRRTM2 was sufficient to saturate presynaptic assembly at contact sites, or because the mechanism whereby the HS contributes to synapse formation may differ between the two ligands.

Finally we tested if CA10 affected synapse formation induced by secreted cerebellins, which lack known HS‐binding sites but bind with high affinity to neurexins containing SS4 (Uemura *et al*, 2010). For this, we used a bead‐based approach, similar to previously described (Matsuda & Yuzaki, 2011). Beads coupled to a monoclonal antibody against the V5‐tag were pre‐incubated with recombinant V5‐Cbln1 and added to DIV8 hippocampal cultures transduced to express FLAG‐tagged CA10 or empty control. After 3 days, neurons were fixed and stained for the synaptic marker synapsin (Fig 7E). Cbln1 induced synapse formation in cultures equally well in the presence or absence of CA10 (Fig 7F), consistent with the notion that Cbln1 does not depend on neurexin HS and predominantly binds non‐HS neurexin in the cerebellum.

These results demonstrate that CA10 can modulate at least some synaptic functions of neurexin. By controlling its HS modification, CA10 may indirectly regulate neurexin–ligand interactions to fine‐tune the properties of specific synapses.

## Discussion

Neurexins are central presynaptic organizers genetically linked to psychiatric and neuropsychiatric disorders in humans. Despite being extensively studied, no unifying function of neurexins has been described. Instead, a model has emerged of neurexins as protein interaction platforms that recruit synapse‐specific repertoires of pre‐ and postsynaptic components by means of their isoform diversity (reviewed in Sudhof, 2017). Here, we study an aspect of how neurexins are molecularly diversified by HS and how this post‐translational modification can be controlled by CA10.

The HS‐modified serine is conserved and located in the stalk region present in all neurexin isoforms and splice variants, but how can this modification be differentially regulated? We have identified a possible mechanism that involves the direct protein–protein interaction between neurexin and CA10: First, we observed that neurexin complexed with CA10 in mouse brains lacked HS (Fig 2I–K). Second, increased levels of CA10 in neurons or HEK293 cells were sufficient to totally block the neurexin HS modification (Fig 2A–H). Third, neurexin mutants to which CA10 cannot bind were HS‐modified also in the presence of CA10 (Fig 3). These findings partially explain our previous observation that exogenous expression of CA10 increases surface levels and shifts the isoform distribution of HA‐tagged Nrxn1 (Sterky *et al*, 2017). Augmented surface levels may reflect increased trafficking of non‐HS neurexin through the secretory pathway, although this remains to be further studied. The CA10–neurexin interaction was previously shown to occur via formation of an intermolecular disulfide between the two proteins (Sterky *et al*, 2017). However, we here found that formation of this bond was dispensable for the ability of CA10 to prevent the HS modification, as this inhibition remained also after the neurexin Cys‐loop (Fig 3A) or the reactive cysteine on CA10 (Fig 3B) had been altered. How can this discrepancy be explained? We believe that a non‐covalent interaction between CA10 and the leucine–valine neurexin residues precede formation of the disulfide bond. This interaction is however weak and thus probably transient, favored by conditions in the secretory pathway, as robust complex between the two proteins without this disulfide was not observed (Sterky *et al*, 2017). When CA10 is overexpressed, this weak interaction may be sufficient to saturate its neurexin‐binding site within the secretory pathway. At the cell surface, however, the proteins dissociate (exemplified in Fig 3G, where cysteine‐mutant CA10 prevented the HS modification, but did not co‐immunoprecipitate with neurexin).

Moreover, we provide mechanistic insights into how CA10 blocks the neurexin HS modification. Using MS (Figs 5 and EV5), we could show that the modified serine is entirely non‐modified in the presence of CA10, indicating that CA10 blocks the very first step in HS biosynthesis: the transfer of xylose from UDP‐xylose to the serine side chain by the xylosyltransferases. The crystal structure of xylosyltransferase 1 has been solved (Briggs & Hohenester, 2018) and has revealed that its substrates require four residues on either side of the serine. The bulky CA10–neurexin complex is unlikely to be accepted as a substrate for the xylosyltransferase. We thus propose that CA10 blocks xylose addition onto neurexins by directly preventing the xylosyltranferase access to the serine by steric hindrance. This idea is supported by the following observations: (i) HS biosynthesis on to GPC1 was unaffected by CA10 (Fig EV2C), excluding the alternative possibility that CA10 directly inhibits xylosyltranferase activity; (ii) residues critical for CA10 binding (Sterky *et al*, 2017) overlap with sequences required the neurexin HS modification (Fig 1); (iii) our NMR data (Fig 4) demonstrates that CA10 engages with residues on both sides of the HS‐modified serine, and (iv) a direct CA10 interaction with neurexin is required to prevent the HS modification (Fig 3A), as CA10 did not block the glycosylation of a neurexin mutant (GIGG) to which CA10 does not bind. These results clearly point to the requirement of a direct interaction between neurexin and CA10. Although we have not been able to directly demonstrate where in the secretory pathway this binding occurs, our results indicate that it must form prior to protein xylosylation. Xylosyltransferases primarily reside in and are active in the *cis‐*Golgi (Nuwayhid *et al*, 1986; Schön *et al*, 2006), but pulse‐chase experiments in permeabilized chondrocytes have shown their activity to begin already in the late ER and continue *en route* to Golgi (Kearns *et al*, 1993; Vertel *et al*, 1993). Taking this into account, we postulate that the CA10‐neurexin interaction must form early in the ER.

The fraction of neurexins carrying the HS modification differs between brain regions (Fig 6) and is almost completely lacking in the cerebellum, where expression levels of CA10 is high (Aspatwar *et al*, 2010; Sterky *et al*, 2017). This demonstrates that the HS chain is not a ubiquitous component of all neurexin complexes and that tunable regulation of HS addition, in addition to alternative promoter use and splicing, can be added to the list of mechanisms generating a diversified palette of neurexins at specific synapses. The neurexin HS chain greatly increases the number of potential neurexin ligands to include a plethora of HS‐binding proteins, but its functional role is not well understood. Previous studies have shown that it cooperates in the binding between neurexins and its canonical *trans*‐synaptic ligands Nlgns and LRRTMs, and may also serve to recruit presynaptic R‐PTPσ in *cis* (Zhang *et al*, 2018; Roppongi *et al*, 2020). A possible scenario is that low affinity interactions between the HS chain and various ligands assist in the assembly of neurexin complexes, which are subsequently stabilized by higher‐affinity protein–protein interactions. However, not all synapses and circuits may require HS‐dependent mechanisms. For example, the low levels of HS in the cerebellum presumably reflects functional specialization of neurexins in the cerebellar circuitry, where most neurexins can be attributed to parallel fibers that engage with Cbln1/GluR‐delta‐2 complexes (Uemura *et al*, 2010; Zhang *et al*, 2015). Extending this notion, differential HS regulation may also confer synapse‐specific properties in subsets of synapses in other brain regions and circuits, where our dissections admittedly provide limited resolution. In support of this we, show that regulation of the neurexin HS modification by CA10 can affect at least some of the synaptic functions of neurexins (Fig 7). We observed that expression of CA10 reduced binding of LRRTM2 to endogenous neurexins on cell surfaces, and attenuated the formation of artificial synapses induced by Nlgn1 in a heparinase‐dependent manner, consistent with the view that the HS chain facilitates binding of these *trans*‐synaptic ligands to the neurexin LNS6 domain (Zhang *et al*, 2018). In addition, synapse formation induced by Cbln1 was unaffected by CA10 (Fig 7E and F). This suggests that Cbln/GluR‐delta complexes do not engage the neurexin HS chain, which is compatible with its low abundance in the cerebellum, although additional experiments *in vivo* will be needed to address this hypothesis. Moreover, future studies should assess whether knockouts for CA10, CA11 and/or other possible ligands to the neurexin Cys‐loop show de‐repressed HS‐neurexin levels in specific brain regions.

**Figure 8:**
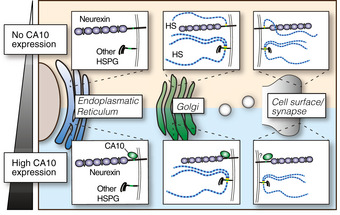
Illustration of how CA10 may control HS specifically on neurexins CA10 binding to neurexin in the ER along the secretory pathway to the cell surface, which sterically hinder the priming xylose addition by xylosyltransferases in the cis‐Golgi. This interaction provides a mechanism to regulate glycan addition at a specific site, to regulate the balance between non‐HS‐ and HS neurexin in a specific cell type without affecting biogenesis of other HSPGs. Control of neurexin HS by CA10 affects the affinities of neurexin to postsynaptic ligands.

The CA10–neurexin interaction in the secretory pathway exemplifies a novel mechanism to regulate site‐specific glycosylation: via a direct protein–protein interaction in the secretory pathway that blocks access of xylosyltransferases to a specific site. HS biosynthesis has previously been found to be regulated at the level of xylose phosphorylation (Wen *et al*, 2014), by differential expression of glycosyltransferases (Roch *et al*, 2010; Condomitti & de Wit, 2018), and even by a post‐transcriptional mechanism (Bornemann *et al*, 2008). These mechanisms are expected to equally affect the synthesis of all HSPGs in the cell. A protein–protein interaction between a proteoglycan and chaperone‐like proteins such as CA10, on the other hand, would only affect a specific GAG attachment site. High expression of CA10 in certain cell types may ensure expression of non‐HS neurexin while maintaining biosynthesis of other HSPGs (Fig 8).

The present work adds another level of complexity to the dynamic regulation of neurexins by a post‐translational mechanism. CA10 engages with neurexin prior to HS synthesis and due to the spatial organization of the secretory pathway, blocks formation of neurexin HS. In this way, CA10 can control the composition of synaptic neurexin complexes.

## Materials and Methods

### Constructs

Constructs to express V5‐ or FLAG‐tagged CA10 and Fc‐tagged Nrxn1β and Nrxn3β variants have been previously described (Sterky *et al*, 2017). To generate HA‐Nrxn1α, an HA tag flanked by GGQ spacers was inserted upstream of stalk residues QTTD (see Fig 4A for stalk sequence) into mouse Nrxn1α (splice‐isoform SS1−, SS2−, SS3+, SS4−, SS5+, SS6+). The HA‐Nrxn1α(S > A) mutant was generated by site‐directed mutagenesis using the QuikChange Lightning Kit (Agilent). Fc‐tagged Nlgn1 and LRRTM2 were generated by inserting the ectodomains of rat *Nlgn1* (lacking A and B inserts) or human *LRRTM2* (HsCD00419164; PlasmID repository at Harvard Medical School), respectively, upstream of a sequence encoding the human Fc in a vector based on pEBMulti (Wako Chemicals). Inserts for cloning were amplified using PrimeStar HS DNA polymerase (Takara) and ligated in vectors by isothermal assembly using the HiFi DNA Assembly kit (NEB). Resulting plasmids were verified by Sanger sequencing. A plasmid encoding HA‐ and FLAG‐tagged GPC1 (HsCD00459620) was obtained from the PlasmID repository at Harvard Medical School.

### Recombinant proteins

Recombinant FLAG/HIS‐tagged CA10 used for NMR experiments was produced by transient transfection of 293F cells in 2 L of Freestyle 293 media using FectoPro transfection reagent (Polyplus). Culture supernatant was harvested and replaced 48 h after transfection, harvested again after 96 h and pooled. The protein was purified on a HisTrap Excel column (GE Healthcare) connected to an ÄktaPure 25 (GE Healthcare) and eluted step‐wise with 100–500 mM imidazole. The eluted protein was buffer‐exchanged to PBS and further concentrated using 10 kDa MWCO spin filters (Millipore). Recombinant V5‐tagged CA10 has been previously described (Sterky *et al*, 2017). Recombinant Fc‐tagged Nlgn1 and LRRTM2 were produced in monolayer HEK293 cells grown in DMEM‐Glutamax (Gibco/Thermo Fisher Scientific) supplemented with 10% (vol/vol) FBS (Sigma‐Aldrich) and transfected using calcium phosphate. Four to 6 h after transfection, the media were replaced by serum‐free FreeStyle 293 media (Gibco/Thermo Fisher Scientific). Media were harvested 3 days after transfection, pre‐concentrated using 10 kDa MWCO spin filters (Millipore) and bound in batch to washed protein A resin (Sepharose 4B; Thermo Fisher Scientific) overnight at 4°C. Beads were packed on gravity‐flow columns and washed twice with 1× HBSS (Gibco/Thermo Fisher Scientific) followed by elution with 0.1 M glycine (pH 2.5). Eluates were immediately neutralized in Tris–HCl (pH 8.0) followed by dialysis into 1× HBSS. Expression and purification of recombinant V5‐ and HIS‐tagged Cbln1 have been previously described (Sterky *et al*, 2017).

### Mouse brain dissection and homogenization

C57BL/6N mice (Taconic) were maintained under conventional conditions with a 12 h light and day cycle in a humidity‐ and temperature‐controlled room. Mice (males and females) were sacrificed under isoflurane anesthesia and indicated brains dissected and snap‐frozen in liquid nitrogen. Animal experiments were approved by an ethics committee and conducted in accordance with Swedish law. Mouse brains used for immunoprecipitations and heparinase digestions were prepared essentially as described (Zhang *et al*, 2018) from 8‐week‐old mice: brain tissue was homogenized in 10× (vol/wt) homogenization buffer (320 mM Sucrose, 4 mM HEPES‐NaOH pH 7.3) supplemented with cOmplete EDTA‐free protease inhibitors (Roche) and 1 mM PMSF using a Potter–Elvehjem tissue grinder (15 strokes, 1,100 rpm). The homogenate was cleared from debris by centrifugation for 10 min at 1,000 × *g* and 4°C. The supernatant was centrifuged for other 10 min at 12,000 × *g* and 4°C, and the pellet washed in resuspension buffer (6 mM Tris pH 8, 0.32 M Sucrose, 1 mM EDTA, 1 mM EGTA, 1 mM DTT and protease inhibitors) and centrifuged for 15 min at 14,500 × *g*. Supernatant was discarded and the final pellet (washed crude synaptosomes) was resuspended in lysis buffer (50 mM Tris pH 7.4, 150 mM NaCl, 1% Triton X‐100, 1 mM EDTA and protease inhibitors), incubated for 30 min at 4°C, and cleared by centrifugation for 30 min at 20,000 × *g*, resulting supernatants were kept. Total protein concentrations of cleared lysates were measured using the BCA kit (Pierce). For co‐immunoprecipitations (Fig 2I), total brain lysates were prepared from 8‐week‐old mice by homogenizing brains in 10× (vol/wt) IP buffer (100 mM NaCl, 4 mM KCl, 2 mM CaCl_2_, MgCl_2_, 20 mM Tris pH 7.4) supplemented with cOmplete EDTA‐free protease inhibitors (Roche) and 1 mM PMSF using a Potter–Elvehjem tissue grinder (15 strokes, 1,100 rpm). Triton X‐100 was added to a final concentration of 1% and incubated for 1 h, followed by centrifugation at 20,000 × *g* for 30 min. Supernatant was kept, and total protein concentration of the cleared lysate was determined for further use in immunoprecipitations.

### Lentiviral production

Lentiviral particles were produced by co‐transfecting HEK293 (see below) with lentiviral expression constructs and third generation packaging plasmids (pRRE, pREV, pVSVG). The media was replaced 4–6 h after transfection to neuronal growth media (see below). Medium was harvested 44–48 h after transfection and cleared from dead cells and debris by centrifugation for 10 min at 1,500 × *g* and 4°C, and the supernatant aliquoted, snap‐frozen in liquid nitrogen, and stored at −80°C until further use.

### Primary mouse cultures

Primary neuron cultures were prepared from P0 to 0.5 pups of C57BL/6N breeding pairs. Cortices and hippocampi were dissected and freed from meninges in cold HBSS (Gibco/Thermo Fisher Scientific; pH 7.4) and (hippocampi only) enzymatically digested [2% papain suspension (Worthington), 2 U/ml DnaseI in HBSS] for 20 min at 37°C. Digested tissue was washed with warm plating medium [MEM (Gibco/Thermo Fisher Scientific) supplemented with 10% FBS (HyClone), 2 mM glutamine and 0.5% glucose] and gently triturated using flame‐polished pasteur pipettes. Dissociated cells were strained through a 70 μm filter and plated on Matrigel (BD Biosciences)‐coated glass cover slips or plastic wells. The media (80% volume) was replaced on the following morning to neuronal growth media [Neurobasal‐A supplemented with 2 mM Glutamine and 1× B27 Supplement (all from Gibco/Thermo Fisher Scientific) and 5% FBS (HyClone)]. Approximately 30 h after plating, 50% of the media volume was replaced and supplemented with arabinose C (Sigma‐Aldrich, 2 mM final concentration). Lentiviral transductions were performed on DIV4‐5 and cells harvested on DIV14‐15. Media was aspirated, and cells were washed 1× with PBS and lysed with Lysis buffer (50 mM Tris pH 7.4, 150 mM NaCl, 1% Triton X‐100, 1 mM EDTA) containing protease inhibitors. After 30‐min incubation on ice, lysates were cleared by centrifugation (10 min, 10,000 × *g*, 4°C) and supernatant saved for further use.

### Biochemical experiments with HEK293 cells

HEK293 cells grown in DMEM‐Glutamax (Gibco/Thermo Fisher Scientific) supplemented with 10% FBS (Sigma‐Aldrich) were transfected with calcium phosphate. Media was replaced with fresh media supplemented with 25 μM chloroquine (Sigma‐Aldrich) 1 h before transfection, and again with fresh media without chloroquine 4–6 h after transfection. Forty‐eight hours after transfection, cells were lysed with RIPA buffer supplemented with protease inhibitors. For co‐secretion assays (Figs 1, 3B and E, and EV1, EV2, EV3), media was replaced by serum‐free FreeStyle 293 media (Gibco/Thermo Fisher Scientific). The media was harvested 48 h after transfection and centrifuged for 10 min, 1,500 × *g*, and 4°C to remove cell debris. Protein A Sepharose (4B; Thermo Fisher Scientific) washed three times in PBS buffer was added to each sample (50 µl) and incubated for 2 h at 4°C. Beads were treated with heparinases as described below. For experiments with membrane‐bound proteins (Figs 2C and D and 3G), HEK293 cells were lysed with RIPA buffer supplemented with protease inhibitors 48 h after transfection. Cell lysates were diluted in IP buffer (100 mM NaCl, 4 mM KCl, 2 mM CaCl_2_, 1 mM MgCl_2_, 20 mM Tris, pH 7.4) and incubated with covalently coupled anti‐HA (Sigma‐Aldrich A2095) or anti‐V5 (Sigma‐Aldrich A7345) beads overnight at 4°C. Beads were treated with heparinases as described below.

### Immunoprecipitations of primary cortical cultures and brain homogenates

Cell lysates or brain homogenates were diluted (equal amount of total protein in the different samples) in IP buffer (100 mM NaCl, 4 mM KCl, 2 mM CaCl_2_, 1 mM MgCl_2_, 20 mM Tris, pH 7.4). For immunoprecipitations from cell lysates of primary cortical cultures (Fig 2F), 5 µg of primary antibody: pan‐neurexin (rabbit, ABN161‐I; Merck) was used. Protein A Sepharose (4B; Thermo Fisher Scientific) washed three times in IP buffer was added to each sample (50 µl) and incubated for 2 h at 4°C. For immunoprecipitations from brain lysates (Figs 6C and 2I) 5 µg of primary antibody: pan‐neurexin (rabbit, ABN161‐I; Merck) or polyclonal antisera directed against recombinant CA10 (rabbit “Hadlai”, Agrisera) were conjugated to 50 µl of Protein A/G magnetic beads (Pierce/Thermo Fisher Scientific) using bis[sulfosuccinimidyl] suberate (BS^3^) (Thermo Fisher Scientific) according to manufacturer’s protocol. Briefly, beads were incubated with the antibodies overnight at 4°C, washed with 0.15 M NaCl and incubated with 5 mM BS^3^ for 30 min at room temperature. The reaction was quenched by adding 50 mM Tris and incubating 15 min at room temperature. Beads were washed with IP buffer and incubated with the samples overnight. Heparinase treatment followed.

### Heparinase treatment

In all cases, heparinase digestions were performed on‐bead following immunoprecipitations. Beads were washed twice with Heparinase buffer (20 mM Tris, 100 mM NaCl, 1.5 mM CaCl_2_) and treated with 1 U/µl of *Flavobacterium heparinum* Heparinase I (Sigma‐Aldrich H2519), Heparinase II (Sigma‐Aldrich H6512), and Heparinase III (Sigma‐Aldrich H8891) for 2 h each at 37°C. Samples were eluted with 35 µl of 2× Laemmli buffer [1×: 10% glycerol, 2% (wt/vol) SDS, 0.1% bromophenol blue, 10 mM EDTA, 60 mM Tris pH 6.8] at 65°C for 10 min, and subsequently analyzed by immunoblotting.

### Immunoblotting and antibodies

Samples (boiled for 5 min) were separated by SDS–PAGE on 4–20% Criterion TGX gels (Bio‐Rad), and transferred to nitrocellulose membranes (GE Healthcare). Membranes were blocked with 5% (wt/vol) nonfat milk (Bio‐Rad) in Tris‐buffered saline with 0.1% Tween‐20 and incubated with primary antibodies overnight at 4°C. The following antibodies were used for immunoblotting: pan‐neurexin1 (rabbit polyclonal, Millipore ABN161; RRID:AB_10917110; used at 1:2,000 dilution), HS stub (mouse monoclonal 3G10; Amsbio; RRID:AB_10892311; used at 1:3,000 dilution), HA (mouse monoclonal HA.11, BioLegend; RRID:AB_2565006; used at 1:2,000 dilution), V5 (mouse monoclonal R960‐25, Thermo Fisher Scientific; RRID:AB_2556564; used at 1:5,000 dilution), FLAG (mouse monoclonal M2, Sigma‐Aldrich, RRID:AB_262044; used at 1:2,000 dilution), actin (mouse monoclonal AC‐74, Sigma‐Aldrich; RRID:AB_476697; used at 1:5,000 dilution), and CA10 (rabbit polyclonal HPA054825, Sigma‐Aldrich; RRID:AB_2682617; used at 1:800 dilution). Near‐infrared 680RD‐ or 800CW‐coupled secondary antibodies (LI‐COR) were used for detection. Membranes were scanned using an Odyssey CLx System (LI‐COR) and quantification of band densities performed using LI‐COR Image Studio software.

### Glycopeptide analysis by mass spectrometry

Secreted Nrxn1β‐Fc expressed alone or in complex with CA10 (Fig EV5A) was concentrated on protein A sepharose beads and subjected to on‐bead heparinase digestion (Fig EV5B), as described above. Samples were eluted in 20 mM HEPES buffer, pH 7.4, containing 2% sodium dodecyl sulfate (SDS) were reduced with 100 mM dithiothreitol (DTT) at 56°C for 30 min. Reduced samples were digested using the filter‐aided sample preparation (FASP) method modified from (Wiśniewski *et al*, 2009). In brief, samples, diluted by addition of 8 M urea, were applied on Nanosep 30k Omega filters (Pall Life Sciences, Port Washington, NY, USA) then 200 µl 8 M urea was used to repeatedly wash away the SDS. Alkylation was performed with 18 mM 2‐iodoacetamide (IAM) for 30 min at room temperature (in the dark), followed by repeated washes with digestion buffer (0.5% sodium deoxycholate, 50 mM TEAB). Asp‐N protease (rAsp‐N, mass Spec Grade, Promega) in digestion buffer was added in a ratio of 1:50 relative to protein amount and samples were incubated at 37°C overnight. Another portion of rAsp‐N was then added and incubated for 3 h. Peptides were collected by centrifugation (10,000 × *g*, 20 min). Collected peptide samples were desalted using Pierce™ Peptide Desalting Spin Columns (Thermo Fischer Scientific) following the manufacturer’s guidelines. The salt free supernatants were dried down and reconstituted in 2% acetonitrile (ACN) in 0.1% formic acid (FA) for LC‐MS analysis. Digested samples were analyzed on an QExactive HF mass spectrometer interfaced with Easy‐nLC1200 liquid chromatography system (Thermo Fisher Scientific). Peptides were trapped on an Acclaim Pepmap 100 C18 trap column (100 μm × 2 cm, particle size 5 μm, Thermo Fischer Scientific) and separated on an in‐house packed analytical column (75 μm × 300 mm, particle size 3 μm, Reprosil‐Pur C18, Dr. Maisch) using a gradient from 5 to 50% B over 75 min, followed by an increase to 100% B for 5 min at a flow of 300 nl/min, where solvent A was 0.2% FA and solvent B was 80% ACN in 0.2% FA. The instrument operated in data‐dependent mode where the precursor ion mass spectra were acquired at a resolution of 120 000, *m*/*z* range 700–1,600. The 10 most intense ions with charge states 2–4 were selected for fragmentation using HCD at collision energy settings of 28. The isolation window was set to 3 *m*/*z* and dynamic exclusion to 20 s. MS2 spectra were recorded at a resolution of 30,000 with maximum injection time set to 110 ms. The acquired data were analyzed using Proteome Discoverer version 2.4 (Thermo Fisher Scientific). Database searches were performed with either Byonic (Protein Metrics) or Sequest as search engines. The data were searched against custom protein database consisting of Swissprot *Homo sapiens* database plus the Nrxn1β‐Fc sequence. Precursor mass tolerance was set to 10 ppm and fragment mass tolerance of 200 millimass units. Asp‐N peptides with up to four missed cleavages were accepted together with variable modification of methionine oxidation and fixed cysteine alkylation. The expected glycosylations were included as variable modifications and were limited to four entries, i.e., Hex, HexNAcHex, XylGalGalGlcA‐H_2_O, and XylGalGalGlcAHexNAcHexA‐H_2_O. Target Decoy was used for PSM validation with the strict FDR threshold of 1%. Identified proteins were filtered at 1% FDR and grouped by sharing the same sequences to minimize redundancy. All glycopeptide identifications were manually evaluated prior to the final assignment of the observed glycosylation forms. The extracted ion chromatogram (EIC) peak intensities were used to determine the glycoform abundances, expressed as percent of total signal for all modified and non‐modified forms.

### Nrxn1 peptide synthesis and purification

Amino acid derivatives, coupling reagents, and resins were procured from Novabiochem, and used without further purification. The peptide (Fig 4A) was synthesized on an INTAVIS MultiPep CF peptide synthesizer using a standard Fmoc‐SPPS protocol on Rink amide MBHA resin (4‐(2′,4′‐dimethoxyphenyl‐Fmoc‐aminomethyl)‐phenoxyacetamido‐norleucyl‐4‐methylbenzhydrylamine) with a loading capacity of 0.23 mmol/g and *N,N,N*′*,N*′‐Tetramethyl‐*O‐*(1*H*‐benzotriazol‐1‐yl)uronium hexafluorophosphate (HBTU, four equivalents relative to loading capacity) as coupling reagents. The base diisopropylethyl amine (DIEA) was used in 2‐fold excess compared with amino acids and coupling reagents. Cleavage from the resin was achieved with 95% trifluoracetic acid (TFA), 2.5% triisopropyl silane and 2.5% water for 3 h. The peptide was precipitated in cold diethyl ether, washed several times and freeze‐dried prior to purification by semi‐preparative HPLC using a Waters 600 system (Waters, Milford, MA, USA) equipped with a C18 column (MultoKrom 100—5 C18, 5 µm particle size, 100 Å pore size, 250 × 20 mm, CS Chromatographie Service, Langerwehe, Germany). The gradient elution system was 0.1% trifluoracetic acid (TFA) in water (eluent A) and 0.1% TFA in acetonitrile (eluent B). The peptides were eluted with a gradient of 15–40% eluent B in 120 min with a flow rate of 8 ml/min. The peaks were detected at 220 nm. Collected fractions were combined, freeze‐dried, and stored at −28°C. Purity of the collected fractions was confirmed by analytical RP‐HPLC (Fig EV4A) on a Waters XC e2695 system (Waters, Milford, MA, USA) employing a Waters PDA 2998 diode array detector equipped with a ISAspher 100‐3 C18 (C18, 3.0 µm particle size, 100 Å pore size, 50 × 4.6 mm, Isera GmbH, Düren, Germany). The peptide was eluted with a gradient of 15–35% eluent B in 10 min at a flow rate of 2 ml/min. Chromatograms were extracted at 214 nm. The molecular weight of the purified peptide was confirmed by ESI mass on a Waters Synapt G2‐Si ESI mass spectrometer equipped with a Waters Acquity UPLC system (Fig EV4B).

### NMR spectroscopy and interaction analysis

NMR spectra were recorded on a Bruker Avance III HD spectrometer at a proton frequency of 800 MHz equipped with a 3 mm CP‐TCI probe. In all NMR spectra, the ^1^H peak of water was used as a chemical shift reference by setting its frequency to 4.7 ppm. All NMR data were processed and analyzed using TopSpin 3.1 (Bruker) and CcpNMR Analysis (Vranken *et al*, 2005). For structure determination of the Nrxn1 stalk, freeze‐dried solid Nrxn1 stalk peptide was used at a final concentration of approximately 2 mM in 90% H_2_O/10% D_2_O. Proton resonance assignment was achieved by a combination of 2D [^1^H,^1^H]‐DQF‐COSY, [^1^H,^1^H]‐TOCSY, [^1^H,^13^C]‐HSQC, and [^1^H,^15^N]‐TROSY (Schulte‐Herbrüggen & Sorensen, 2000) (Table EV2). Distance constraints were extracted from [^1^H,^1^H]‐NOESY spectrum acquired with a mixing time of 120 ms and a recycle delay time of 1.8 s. Upper limit distance constraints were calibrated according to their volume in the NOESY spectrum and the volume of geminal protons was used for peak intensity calibration. Torsion angle constraints were obtained from ^1^H and ^13^C chemical shift analysis using DANGLE and ^3^J_NHHa_‐coupling constants (Cheung *et al*, 2010). Structure calculations and refinements were performed with YASARA structure (Harjes *et al*, 2006; Krieger & Vriend, 2014; Krieger & Vriend, 2015). The 10 structures with the lowest energy were selected to represent the NMR solution structures (Fig 4B, Table EV1).

For analysis of interaction, the Nrxn1 stalk peptide and FLAG‐tagged recombinant CA10 were analyzed at peptide:protein ratios of 10:1 and 2:1 (90 µM Nrxn1, 9 µM CA10 and 90 µM Nrxn1, 45 µM Ca10, respectively) in d10‐DTT (0.5 mM) containing phosphate buffer (50 mM, pH 7.4, 10% D_2_O). [^1^H,^1^H]‐TOCSY and [^1^H,^13^C]‐HSQC spectra were analyzed regarding chemical shift changes of the peptide ligand in presence of CA10.

### Immunocytochemistry

Cells were fixed in 4% paraformaldehyde, 4% sucrose, 0.1 M PBS for 15 min at room temperature, then permeabilized with 0.1% (*v*/*v*) Triton X‐100 in PBS for 10 min and blocked in IF blocking solution (2% goat serum, 1% BSA in PBS) for 1 h, all steps at room temperature. Primary antibodies SV2 (mouse, clone SV2‐c; Developmental Studies Hybridoma Bank; RRID:AB_2315387; used at 1:500 dilution), synapsin (rabbit antisera E028; RRID:AB_2315400; used at 1:1,000 dilution) and MAP2 (chicken, 2Bscientific; RRID:AB_2138173; used at 1:2,000 dilution) were incubated over night at 4°C in IF blocking solution, washed, and incubated with goat‐anti‐chicken Alexa‐568 (1:1,000 dilution) and goat‐anti‐mouse Alexa‐633 (1:600 dilution) secondaries (Thermo Fisher Scientific) for 1 h at room temperature. DAPI (Sigma‐Aldrich, D9542) was used together with secondary antibodies. Cells were washed, briefly rinsed in distilled water and mounted with Prolong Gold Anti‐Fade mounting medium (Thermo Fisher Scientific, P36934) and allowed to dry completely.

### Artificial synapse formation assay

Lentiviruses expressing CA10‐FLAG or control (empty vector) were used to transduce hippocampal neurons grown on DIV4. HEK293 cells transiently transfected to express GFP, Nlgn1‐YFP, LRRTM2‐YFP, or TrkC‐YFP, respectively, were added on top of the cultures on DIV8. 24 h later, cells were fixed and stained as described above. For heparinase treatment, neurons on coverslips were incubated with 0.4 U/ml of heparinase I, II, and III in conditioned medium for two and half hours prior to addition of HEK293 cells. Cells were imaged using an A1plus confocal system (Nikon Instruments) with a 40×/1.15NA water immersion objective. Maximum intensity projection in z was analyzed for SV2‐immunoreactive signal intensity on regions of interest defined by GFP‐positive cells using NIS‐Elements AR software (v. 5.21.01, Nikon Instruments). For bead‐based artificial synapse formation, 10 μg of washed anti‐V5 coupled magnetic beads (MBL) were incubated with 0.6 μg V5‐tagged Cbln1 in 200 μl DPBS containing 1% BSA. After 1 h at room temperature shaking at 600 rpm, beads were washed once in DPBS + 1% BSA and an equivalent of 1.25 μg of beads/cover slip added to DIV8 primary hippocampal neurons. The neurons were fixed 72 h later and stained for synapsin and MAP2, as described above. Single confocal planes were acquired using an A1plus confocal system (Nikon Instruments) with a 40×/1.15NA water immersion objective and analyzed for synapsin intensity on beads.

### Cell surface binding assay

Lentiviruses expressing CA10‐FLAG or control (empty vector) were added to hippocampal neurons grown in 96‐well plates on DIV4. On DIV10, cells were washed with HEPES bath solution (140 mM NaCl, 4 mM KCl, 2 mM CaCl_2_, 1 mM MgCl_2_, 10 mM glucose, and 10 mM HEPES pH 7.4). Recombinant Nlgn1‐Fc and LRRTM2‐Fc resuspended in HEPES bath solution at concentrations of 0, 25, 50, 75, and 100 nM were added to the wells and incubated for 1 h at room temperature. Cells were fixed and stained as described above for Nrxn1α (rabbit; Frontier Institute; RRID:AB_2571817; used at 1:1,000 dilution) and MAP2 (chicken, 2Bscientific; RRID:AB_2138173; used at 1:2,000 dilution). Secondary antibodies were goat‐anti‐rabbit Alexa‐488, goat‐anti‐chicken Alexa‐633, and goat‐anti‐human Alexa‐568 (all at 1:1,000 dilutions, Thermo Fisher Scientific). Cells were visualized by confocal microscopy using a Nikon EclipseTi confocal A1plus. Images were acquired using a 20×/0.75NA objective on an A1plus confocal system (Nikon Instruments). MAP2 stained area was used to create a binary image and quantify the mean fluorescence intensity of Fc‐Nlgn1 or Fc‐LRRTM2, respectively, normalized by the mean intensity of immunolabeled Nrxn1α in the same areas using NIS‐Elements AR software (v. 5.21.01, Nikon Instruments).

### Quantifications and statistical analysis

Statistical analyses were performed using GraphPad Prism (v. 8) using tests indicated in the corresponding figure legends. Normal distribution was assessed by QQ plots (most datasets were underpowered for formal normality tests). For datasets with apparent non‐normal distribution, non‐parametric Mann–Whitney tests were performed. In other experiments, one‐ or two‐way ANOVA with Holm–Sidak’s tests were performed. Binding curves were analyzed by non‐linear regression. All data are reported as the mean ± standard error of the mean (SEM) with the number of *n* (biological replicates) indicated in each figure legend. For all microscopy experiments, coding of samples blinded the experimenter to the condition analyzed.

## Author contributions

Study conception: LM‐G and FHS. Experiment plan: LM‐G, DT, AAT, and FHS. Experiments: LM‐G, DT, DK, EM, and FHS. Manuscript writing: LM‐G, DT, and FHS.

## Conflict of interest

The authors declare that they have no conflict of interest.

## Supporting information



Appendix

Expanded View Figures PDF

Table EV1

Table EV2

Review Process File

## Data Availability

Source data have been posted in the Dryad repository and are accessible at https://doi.org/10.5061/dryad.z612jm69p.
