# Supplementary material for: CA10 regulates neurexin heparan sulfate addition via a direct binding in the secretory pathway
Source: EMBO Rep. 2021 Feb 15;22(4):e51349. doi: 10.15252/embr.202051349 (PMC8024894; doi:10.15252/embr.202051349)
Supplement: Supplementary file 1 — Appendix [file EMBR-22-e51349-s004.pdf]

CA10 Regulates Neurexin Heparan Sulfate Addition via a Direct Binding in the Secretory Pathway - APPENDIX

Table of content

Appendix Figure S1 (related to Fig 7A-B)……………………………………...page 2

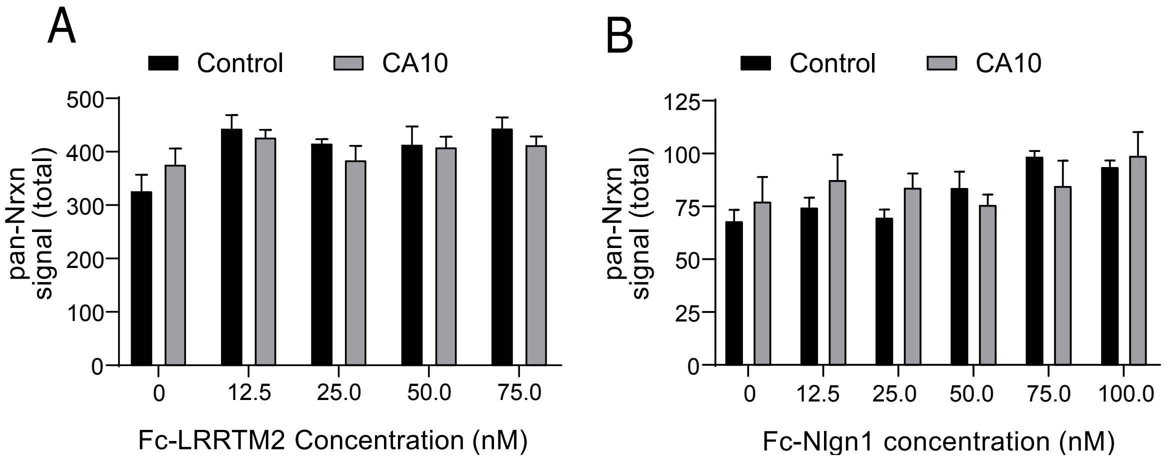

Appendix Figure S1 (related to Fig 7A-B). Total neurexin levels in cell-surface binding experiments, assessed by immunostainings using a polyclonal antibody directed against Nrxn1α but reactive to all neurexin isoforms. Data shown as mean ±SEM of 4 biological replicates.
