# Supplementary material for: CA10 regulates neurexin heparan sulfate addition via a direct binding in the secretory pathway
Source: EMBO Rep. 2021 Feb 15;22(4):e51349. doi: 10.15252/embr.202051349 (PMC8024894; doi:10.15252/embr.202051349)
Supplement: Supplementary file 2 — Expanded View Figures PDF [file EMBR-22-e51349-s003.pdf]

Expanded View Figures

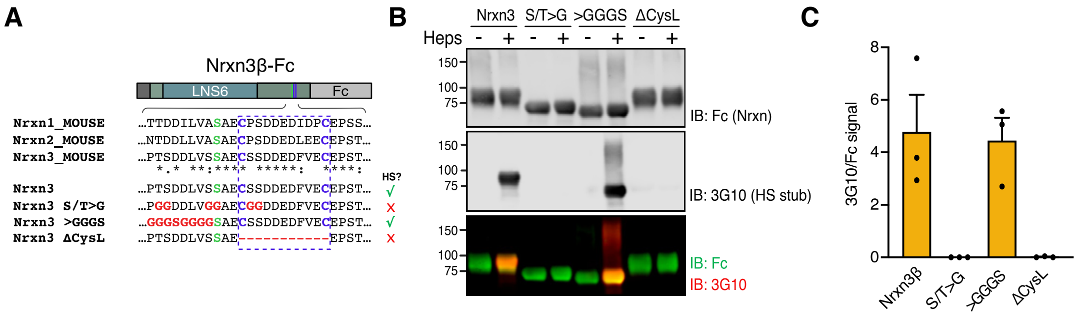

Figure EV1. Analysis of Nrxn3 sequences required for its HS addition (related to Fig 1).

A Sequences of secreted Fc-tagged Nrxn3β variants used. Mutated residues are shown in red, the HS-modified serine in green, and cysteines forming the Cys-loop in blue. Asterisks, colons, and periods indicate fully, strongly, or weakly conserved residues, respectively. Right column ('HS?') summarizes the results from (B, C).

B Representative immunoblot of Nrxn3β-Fc variants harvested from HEK293 cell media and subjected to heparinase treatment ("Heps"). Samples were analyzed by reducing SDS–PAGE and immunoblotting with antibodies against the Fc tag (Nrxn) and the 3G10 epitope (for the HS stub).

C Quantification of 3G10 (reflecting HS) signal normalized to that of Fc (Nrxn). Bar graph shows means ± SEM of 3 independent experiments.

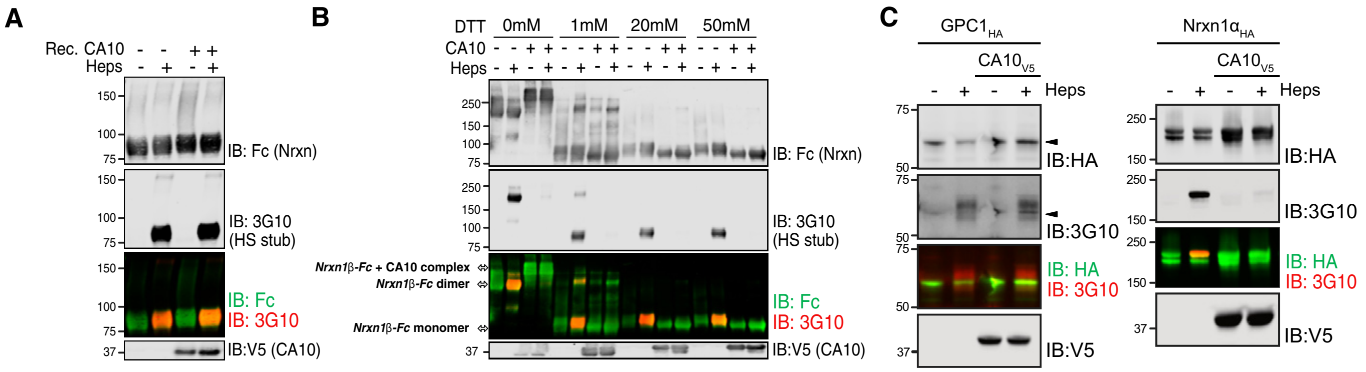

Figure EV2. Heparinase digestion and detection of the HS stub is not inhibited by CA10 and prevention of HS addition by CA10 is specific for neurexins (related to Fig 2).

A Fc-tagged Nrxn1β was captured on protein A beads and digested with heparinases ("Heps") with or without recombinant V5-tagged CA10 (3.5 μM final concentration). Samples were analyzed by SDS–PAGE under reducing conditions, followed by immunoblotting against the Fc tag and the HS stub (3G10).

B Fc-tagged Nrxn1β expressed in HEK293 cells, alone or together with CA10-V5, were captured on protein A beads and treated with heparinases ("Heps") in the presence of indicated concentrations of DTT (to dissociate the Nrxn1β-CA10 complex). Samples were analyzed by SDS–PAGE under non-reducing conditions, followed by immunoblotting against the Fc tag and the HS stub (3G10). DTT at a concentration of 20 mM fully dissociated the CA10- Nrxn1β complex without inhibiting heparinase activities.

C HA-tagged GPC1 (left) or HA-Nrxn1α, processed in parallel as a control (right), were expressed alone or together with V5-tagged CA10 in HEK293 cells, immunoprecipitated for HA and subjected to heparinase treatment ("Heps") to reveal HS-carrying isoforms (similar to the experiment outlined in Fig 2A). Samples were analyzed by immunoblotting with antibodies against HA, the 3G10 epitope (for the HS stub) and V5. Non-HS GPC1 is marked by an arrowhead, while HS-containing GPC1 is not visible prior to heparinase treatment (Wen *et al*, 2014).

© 2021 The Authors

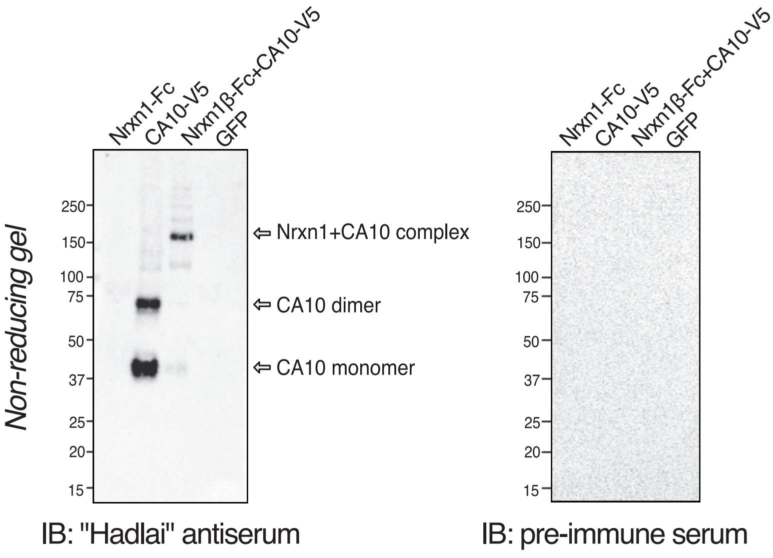

Figure EV3. Characterization of the Nrxn1-CA10 antiserum (related to Fig 3).

Soluble Nrxn1γ -Fc, CA10-V5 and both were expressed in HEK293 cells. Media was analyzed by non-reducing SDS–PAGE and blotted with “Hadlai” antiserum raised against recombinant covalent Nrxn1γ-CA10 complex or pre-immune serum from the same rabbit. The antiserum recognizes CA10 and the Nrxn1-CA10 complex, but not Nrxn1γ alone.

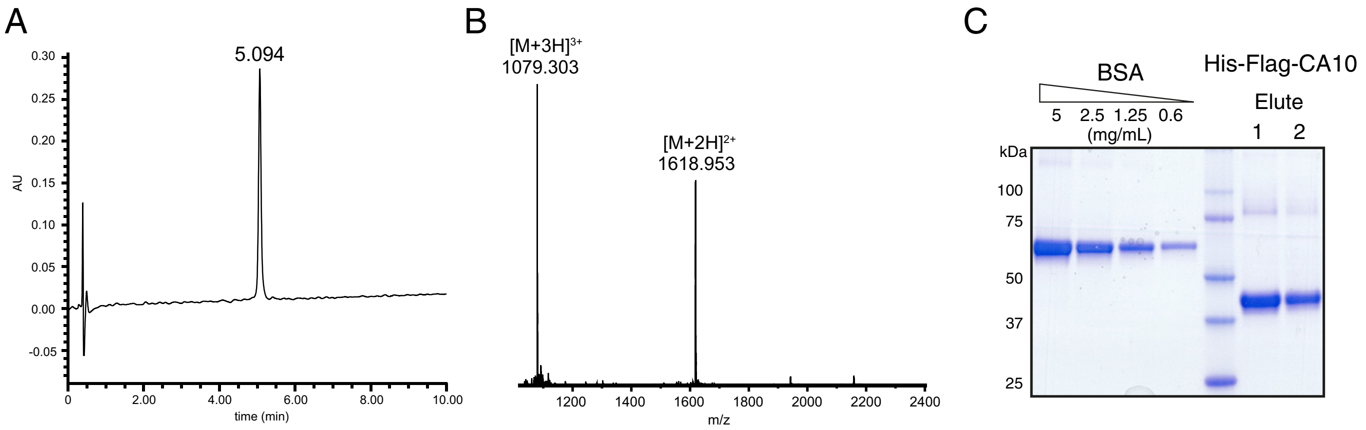

Figure EV4. Analytical characterization of recombinant CA10 and the Nrxn1 stalk peptide (related to Fig 4).

A Analytical HPLC chromatogram of the purified Nrxn1 stalk peptide.
B ESI mass spectrum of the purified Nrxn1 stalk peptide. The calculated average mass is 3,237.35 $[M + H]^{+}$.
C Coomassie-stained gel of recombinant HIS-FLAG-tagged CA10, separated under reducing conditions.

Figure EV5. MS/MS spectra of peptides from recombinant Nrxn1 expressed with or without CA10 (related to Fig 5).

A, B SDS–PAGE gel stained for (A) total protein and (B) immunoblot of the replicate experiments used for MS/MS analysis.
C Fragment spectrum of the unmodified DDILVASAECPSDDE peptide, precursor at *m/z* 818.338 ($[M + 2H]^{2+}$).
D Fragment spectrum of the DDILVASAECPSDDE+ HexNAc, precursor at *m/z* 919.878 ($[M + 2H]^{2+}$).
E Fragment spectrum of the DDILVASAECPSDDE+ HexNAcHex, precursor at *m/z* 1,000.904 ($[M + 2H]^{2+}$).
F Fragment spectrum of the DDILVASAECPSDDE+ XylGalGalGlcA-H_2O, precursor at *m/z* 1,125.423 ($[M + 2H]^{2+}$).
G Table with theoretical MH and retention times (RT) of each glycopeptide, and % total occupancy of each replicate in the control and CA10 samples (values represented in Fig 5D).

Data information: All fragment spectra (C–F) display high similarity with the same major peptide fragments being observed, confirming the peptide identities. The *O*-glycosylated peptides further displayed oxonium ions associated with mucin-type *O*-GalNAc glycosylation.

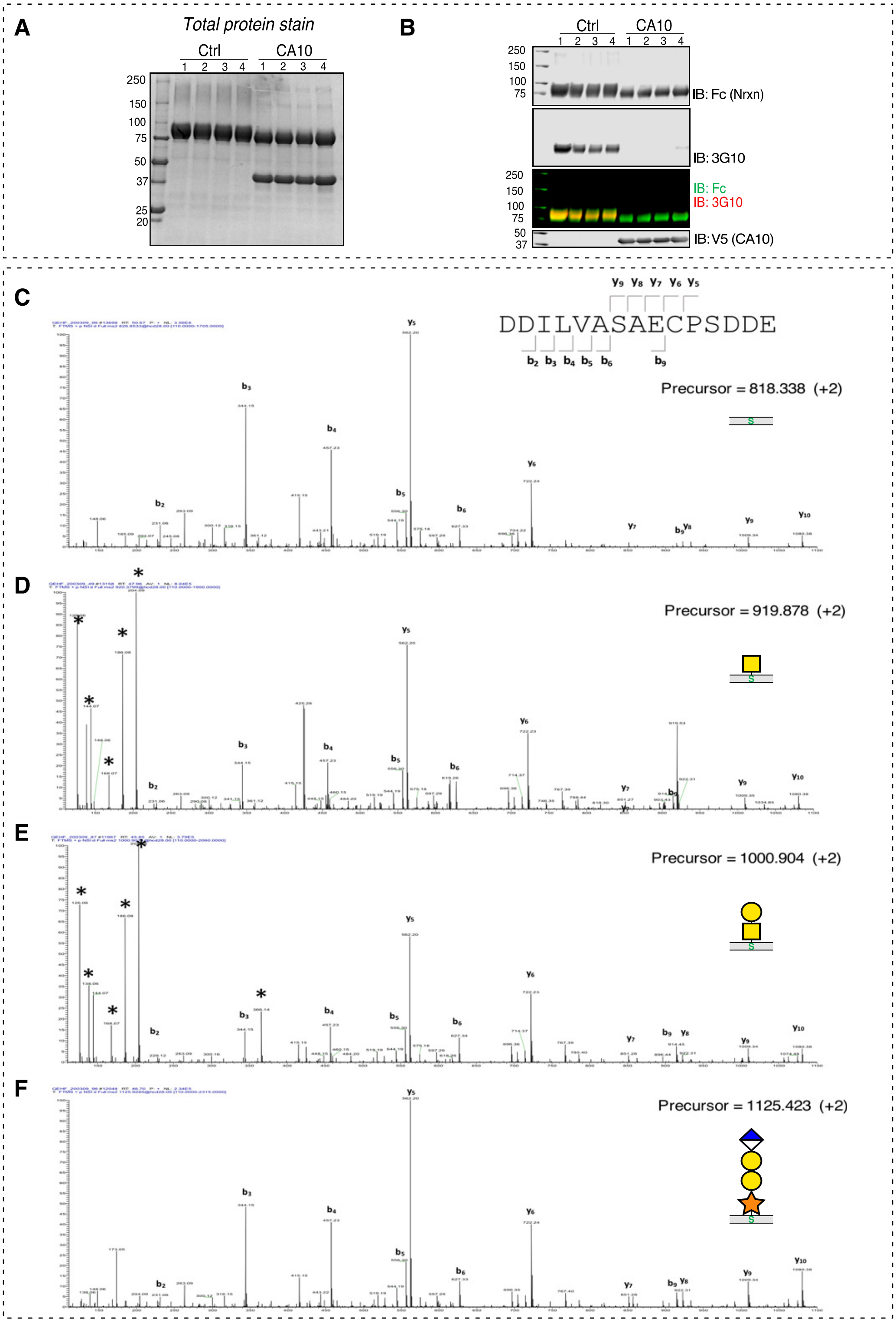

G

Protein / Peptide	Glycosylation	Theo. MH [Da]	RT [min]	Control (% of total)					CA10 (% of total)				
				1	2	3	4	Average	1	2	3	4	Average
[T] .DDLLVASAECPSDDE.[D]	NG	1635.6690	51.0	94.1	93.9	93.4	93.9	93.8	100.0	100.0	100.0	100.0	100.0
[T] .DDLLVASAECPSDDE.[D]	HexNAc	1838.7484	47.8	2.2	2.8	2.7	2.3	2.5	0.0	0.0	0.0	0.0	0.0
[T] .DDLLVASAECPSDDE.[D]	HexNAcHex	2000.8012	46.0	2.2	1.9	2.3	2.4	2.2	0.0	0.0	0.0	0.0	0.0
[T] .DDLLVASAECPSDDE.[D]	XylHexHexDHexA	2249.8385	46.8	1.4	1.4	1.6	1.4	1.5	0.0	0.0	0.0	0.0	0.0

Figure EV5.

© 2021 The Authors
