# Supplementary material for: CA10 regulates neurexin heparan sulfate addition via a direct binding in the secretory pathway
Source: EMBO Rep. 2021 Feb 15;22(4):e51349. doi: 10.15252/embr.202051349 (PMC8024894; doi:10.15252/embr.202051349)
Supplement: Supplementary file 3 — Table EV1 [file EMBR-22-e51349-s002.docx]

Table EV1. Energy and structural statistics of the NMR ensembles of Nrxn1 stalk (10 lowest energy structures). Maximum distance restraints violations <0.1Å.

|  | **Nrxn1 stalk** |
| --- | --- |
| Distance restraints | 146 |
| Dihedral angle restraints | 54 |
| 3J couplings | 3 |
| **Structure statistics** |  |
| Violations | in kJ/mol |
| Total restraint violation energy | 1.35 |
| Distance restraints | 1.32 |
| Dihedral angle restraints | 0.03 |
| Total Force field energy (Yasara) | -12334 |
| Internal solute energy | -4257455 |
| Electrostatic solv. energy | -8068 |
| Van der Waals solv. energy | -8.54 |
| Mean RMSD (Å) | Mean (s.d.) |
| Backbone global | 2.95 (± 0.603) |
| Backbone residues 12-24 | 1.80 (± 0.635) |
| Heavy atoms | 3.15 (± 0.563) |
