# Supplementary figures and images for: CA10 regulates neurexin heparan sulfate addition via a direct binding in the secretory pathway
Source: EMBO Rep. 2021 Feb 15;22(4):e51349. doi: 10.15252/embr.202051349 (PMC8024894; doi:10.15252/embr.202051349)

Table EV2. 1H and 13C NMR spectroscopic data of Nrxn1 stalk (90% H2O / 10% D2O, 298 K).


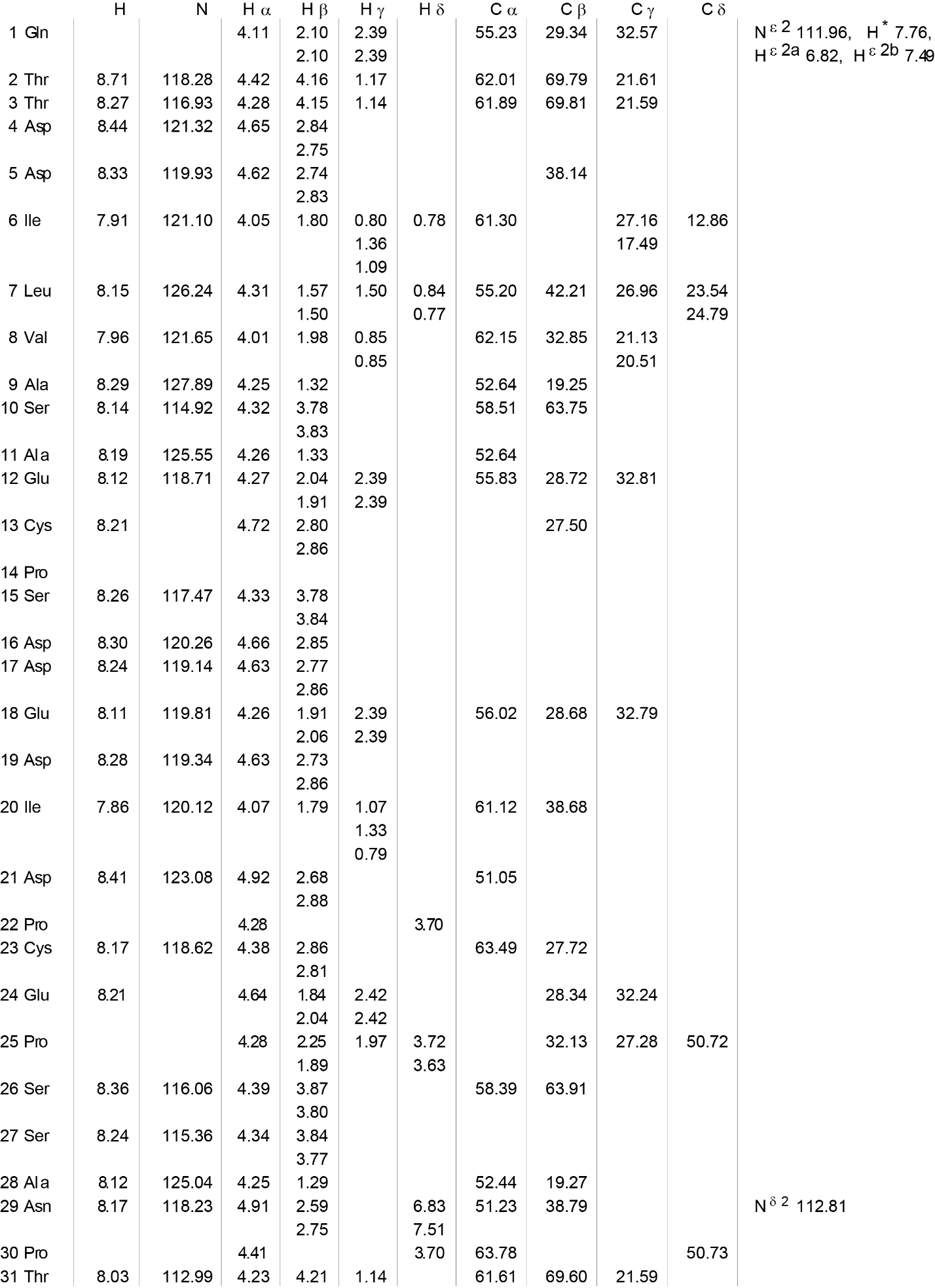

Supplement: Supplementary file 4 — Table EV2 [file EMBR-22-e51349-s005.docx]
