# Supplementary material for: CA10 regulates neurexin heparan sulfate addition via a direct binding in the secretory pathway
Source: EMBO Rep. 2021 Feb 15;22(4):e51349. doi: 10.15252/embr.202051349 (PMC8024894; doi:10.15252/embr.202051349)
Supplement: Supplementary file 5 — Review Process File [file EMBR-22-e51349-s001.pdf]

EMBO reports

CA10 Regulates Neurexin Heparan Sulfate Addition via a Direct Binding in the Secretory Pathway

Laia Montoliu Gaya, Daniel Tietze, Debora Kaminski, Ekaterina Mirgorodskaya, Alesia Tietze, and Fredrik Sterky
DOI: 10.15252/embr.202051349

Corresponding author(s): Fredrik Sterky (fredrik.sterky@gu.se)

Review Timeline:

Submission Date:	17th Jul 20
Editorial Decision:	26th Aug 20
Revision Received:	24th Nov 20
Editorial Decision:	18th Dec 20
Revision Received:	21st Dec 20
Accepted:	13th Jan 21

Editor: Martina Rembold

Transaction Report:

(Note: With the exception of the correction of typographical or spelling errors that could be a source of ambiguity, letters and reports are not edited. Depending on transfer agreements, referee reports obtained elsewhere may or may not be included in this compilation. Referee reports are anonymous unless the Referee chooses to sign their reports.)

1st Editorial Decision 26th Aug 2020

Dear Prof. Sterky

Thank you for the submission of your research manuscript to our journal. We have now received the full set of referee reports that is copied below.

As you will see, the referees acknowledge that the findings are potentially interesting but they also have a number of suggestions for how the study should be strengthened, which should be addressed in a revision. It would certainly strengthen the paper if some data on trans-synaptic Nrxn-ligand interactions that do not depend on HS could be added (referee 2, minor point 7). Also loss-of-function data would be a valuable addition but are not strictly required (referee 2, minor point 6). Also the function and structure of the heparan sulfate on neurexin can be discussed and explained in the text (referee 1, point 1 and 4). All control experiments need to be provided and the statistical evaluation needs to be revisited as suggested by referee 3.

Given these constructive comments, we would like to invite you to revise your manuscript with the understanding that the referee concerns (as detailed above and in their reports) must be fully addressed and their suggestions taken on board. Please address all referee concerns in a complete point-by-point response. Acceptance of the manuscript will depend on a positive outcome of a second round of review. It is EMBO reports policy to allow a single round of revision only and acceptance or rejection of the manuscript will therefore depend on the completeness of your responses included in the next, final version of the manuscript.

We invite you to submit your manuscript within three months of a request for revision. This would be November 26th in your case. Yet, given the current COVID-19 related lockdowns of laboratories, we have extended the revision time for all research manuscripts under our scooping protection to allow for the extra time required to address essential experimental issues. Please contact us to discuss the time needed and the revisions further.

IMPORTANT NOTE: we perform an initial quality control of all revised manuscripts before re-review. Your manuscript will FAIL this control and the handling will be DELAYED if the following APPLIES:

1) A data availability section is missing.
2) Your manuscript contains error bars based on n=2. Please use scatter blots showing the individual datapoints in these cases. The use of statistical tests needs to be justified.

When submitting your revised manuscript, please carefully review the instructions that follow below. Failure to include requested items will delay the evaluation of your revision.

Please note that for all articles published beginning 1 July 2020, the EMBO Reports reference style will change to the Harvard style for all article types. Details and examples are provided at https://www.embopress.org/page/journal/14693178/authorguide#referencesformat

When submitting your revised manuscript, we will require:

1) a .docx formatted version of the manuscript text (including legends for main figures, EV figures and tables). Please make sure that the changes are highlighted to be clearly visible.

2) individual production quality figure files as .eps, .tif, .jpg (one file per figure).
Please download our Figure Preparation Guidelines (figure preparation pdf) from our Author Guidelines pages https://www.embopress.org/page/journal/14693178/authorguide for more info on how to prepare your figures.

3) a .docx formatted letter INCLUDING the reviewers' reports and your detailed point-by-point responses to their comments. As part of the EMBO Press transparent editorial process, the point-by-point response is part of the Review Process File (RPF), which will be published alongside your paper.

4) a complete author checklist, which you can download from our author guidelines (). Please insert information in the checklist that is also reflected in the manuscript. The completed author checklist will also be part of the RPF.

5) Please note that all corresponding authors are required to supply an ORCID ID for their name upon submission of a revised manuscript (). Please find instructions on how to link your ORCID ID to your account in our manuscript tracking system in our Author guidelines
()

6) We replaced Supplementary Information with Expanded View (EV) Figures and Tables that are collapsible/expandable online. A maximum of 5 EV Figures can be typeset. EV Figures should be cited as 'Figure EV1, Figure EV2" etc... in the text and their respective legends should be included in the main text after the legends of regular figures.

- For the figures that you do NOT wish to display as Expanded View figures, they should be bundled together with their legends in a single PDF file called *Appendix*, which should start with a short Table of Content. Appendix figures should be referred to in the main text as: "Appendix Figure S1, Appendix Figure S2" etc. See detailed instructions regarding expanded view here:

- Additional Tables/Datasets should be labeled and referred to as Table EV1, Dataset EV1, etc. Legends have to be provided in a separate tab in case of .xls files. Alternatively, the legend can be supplied as a separate text file (README) and zipped together with the Table/Dataset file.

7) Please note that a Data Availability section at the end of Materials and Methods is now mandatory. In case you have no data that requires deposition in a public database, please state so instead of refereeing to the database.
See also < https://www.embopress.org/page/journal/14693178/authorguide#dataavailability>).
Please note that the Data Availability Section is restricted to new primary data that are part of this study.

8) We would also encourage you to include the source data for figure panels that show essential data. Numerical data should be provided as individual .xls or .csv files (including a tab describing the data). For blots or microscopy, uncropped images should be submitted (using a zip archive if multiple images need to be supplied for one panel). Additional information on source data and instruction on how to label the files are available .

9) Our journal encourages inclusion of *data citations in the reference list* to directly cite datasets that were re-used and obtained from public databases. Data citations in the article text are distinct

from normal bibliographical citations and should directly link to the database records from which the data can be accessed. In the main text, data citations are formatted as follows: "Data ref: Smith et al, 2001" or "Data ref: NCBI Sequence Read Archive PRJNA342805, 2017". In the Reference list, data citations must be labeled with "[DATASET]". A data reference must provide the database name, accession number/identifiers and a resolvable link to the landing page from which the data can be accessed at the end of the reference. Further instructions are available at .

10) Regarding data quantification:
- Please ensure to specify the name of the statistical test used to generate error bars and P values, the number (n) of independent experiments (please specify technical or biological replicates) underlying each data point and the test used to calculate p-values in each figure legend. Discussion of statistical methodology can be reported in the materials and methods section, but figure legends should contain a basic description of n, P and the test applied.
- Graphs must include a description of the bars and the error bars (s.d., s.e.m.).
- Please also include scale bars in all microscopy images.

11) As part of the EMBO publication's Transparent Editorial Process, EMBO reports publishes online a Review Process File to accompany accepted manuscripts. This File will be published in conjunction with your paper and will include the referee reports, your point-by-point response and all pertinent correspondence relating to the manuscript.

You are able to opt out of this by letting the editorial office know (emboreports@embo.org). If you do opt out, the Review Process File link will point to the following statement: "No Review Process File is available with this article, as the authors have chosen not to make the review process public in this case."

We would also welcome the submission of cover suggestions, or motifs to be used by our Graphics Illustrator in designing a cover.

I look forward to seeing a revised version of your manuscript when it is ready. Please let me know if you have questions or comments regarding the revision.

Yours sincerely,

Martina Rembold, PhD
Editor
EMBO reports

****

Referee #1:

EMBOR-2020-51349V1
The paper CA10 Regulates Neurexin Heparan Sulfate Modification via a Direct Binding in the Secretory Pathway by Gaya et al is an interesting and relevant paper. The main finding is that CA10 interacts specifically with the stalk region of neurexin thereby blocking the generation of heparan sulfate. To strengthen the paper the function of the heparan sulfate modified neurexin could be better defined. Many of my suggestion may be too large to include in this paper and could be included in next paper more directed to heparan sulfate.

Major points
1. The paper would be strengthened of the function and structure of the heparan sulfate on neurexin could included. Heparan sulfate interacts with many cytokines and growth factors with clear effects on neurons.
2. The authors claim that the reason for elimination of heparan sulfate is that the serin motif is blocked. Another possibility is that the xylosyltransferases could be blocked. This should at least be discussed
3. CA10 blocks both heparan sulfate and the small HexNAc oligosaccharides. Which of these saccharides are of importance for the neurexin glyco complex
4. The extra bands emerging after heparinase digestion in Fig 2 should be explained. Are other heparan sulfate proteoglycan part of the complex or are they just impurities. The core size of these proteoglycans exclude syndecans glypicans and perlecan. Does these proteoglycan play any role in neurexin synapse function. complex
5. The functional modulation of synapses by CA10 is interesting and clear för NLG1. However the conclusion of effect on TrkC is not clearly shown in fig 7C. There is a clear read staining on the control however no red staining after treatment with CA10. Please explain your conclusion and improve the figure. More serious is that the effect can not be attributed to heparan sulfate in the present stage. Control with heparinase digestion must be performed. As pointed out in point 1 effect of heparan sulfate binding cytokines on synapse formations should be of interest.
6. Do you know where the CA10- neurexin complex is formed in ER or in any of the Golgi compartment. It could however be compared with the xyloxylation of proteoglycans which occur in rough ER https://doi.org/10.3109/03008208408992780

Minor points
1. The title is unclear. Do the authors mean modified neurexin or modified heparan sulfate
2. What does the author mean by HS modification
3. What is HA tag? Does it have any relation to Hyaluronan
4. Fig 2B. Why is HA-Nrxn1alfa+Ca10-V5 smaller than HA-Nrxn1alfa
5. Where is 6F
6. Figure 8. Can you really state that CA10 not affects the formation of other proteoglycans. Remove glypicans and use heparan sulfate proteoglycans. See point 4 on major suggestions.
7. What does the stars indicate in some of the figures and why are the bands stronger in Figure 2 in the presence of CA10

Referee #2:

In this study, Gaya and colleagues investigate the role of the recently identified neurexin (Nrxn) ligand CA10 in regulating heparan sulfate (HS) modification of Nrxn. The authors perform systematic binding analyses to determine that CA10 prevents HS-modification of Nrxn in cultured cells, cultured neurons and in the brain in vivo. The authors show that this effect of CA10 does not require the formation of a disulfide intermolecular bond between CA10 and Nrxn, but relies on non-covalent interactions of CA10 with Nrxn. NMR experiments demonstrate that CA10 binding affects residues surrounding the HS-modified serine in Nrxn, but does not affect the HS-modified serine itself. Altogether these experiments demonstrate that CA10 blocks HS-modification of Nrxn via a direct interaction and likely prevent HS biosynthesis by steric hindrance. At the brain regional level,

the presence of HS-modified Nrxn seems to negatively correlate with the levels of CA10, consistent with the notion that CA10 blocks HS-modification. Functionally, expression of CA10 in neurons reduces the synaptogenic effect of Nlgn1 expressed in heterologous cells, which has previously been demonstrated to depend in part on Nrxn-HS. Together the results show that regulation of HS-modification of Nrxn by CA10 indirectly controls trans-synaptic Nrxn-ligand binding.

This is an interesting study that provides new insight in the regulation of the Nrxn-HS modification. Both CA10 as a Nrxn ligand and HS-modification of Nrxns have only recently been identified. The paper provides a solid analysis of the CA10-Nrxn interaction and its effect on HS-modification of Nrxn. Furthermore, the study identifies a novel regulatory mechanism by which protein-protein interactions in the secretory pathway can control HS modification of a specific target protein, as opposed to other regulatory mechanism that globally affect proteoglycans in the cell.

Major
1. The authors convincingly show that the intermolecular disulfide bond is not required for CA10 blocking HS-modification of Nrxn. The role of the non-covalent interaction is less clear to me. The Nrxn GIGG mutant does not bind CA10 and is HS-modified. The DIRR mutant however also does not bind CA10 but is not HS-modified (Figure 1B, 3A and Sterky et al., PNAS 2017). How can the non-binding mutant prevent HS-modification? This should be experimentally addressed or explained.
2. What was the rationale for not testing LRRTM2 in the coculture assay shown in Figure 7C and 7D? Based on the Scatchard analysis in Figure 7A, the effect of CA10 expression is expected to have a stronger effect on LRRTM2-mediated synaptogenesis than on Nlgn1. This data, even when negative, should be included. The effect of HS on LRRTM2-mediated synaptogenesis in coculture assays may vary depending on the heterologous cell line used (compare Zhang et al., Cell 2018 and Condomitti et al., Neuron 2018).
3. Throughout the paper, the statistics are not clearly described, which makes it difficult to interpret the data. Major points that should receive attention include 1) The authors only use student's T tests or one or two-way ANOVA's with posthoc Bonferroni tests. These tests assume normal distribution of the data and (in the case of the ANOVA's with Bonferroni tests) homogeneity of variance. However, it is unclear from the Material and Methods section if the data was tested for normality and homogeneity of variance. Violation of the assumptions for these tests may result in inflated type 1 error rates. 2) In addition, for Figure 7A and 7B, the authors use a two-way ANOVA. First, this test assumes the independent variables to be categorical, but the "Concentration" variable is continuous. An ANCOVA test should be a better solution for this data. Second, the authors do not describe any posthoc tests on these data. Therefore, it is unjustified to claim that LRRTM2 or Nlgn1 binding is different between the control and CA10 conditions. 3) In multiple cases throughout the paper, statistical tests are not described. For example, in figure 2E and 2H, T tests were used to test the non-digested conditions, but it is unclear how the heparinase conditions were tested. Though by eye the results in these conditions do not seem to differ, it is still unjustified to claim that no difference is observed here without reporting statistical tests. It is highly recommended to note exact p-values for non-significant data in the figure legends as they will support multiple results in the paper.

Minor
1. Page 4 "This cysteine can form an intramolecular disulfide between CA10 and the N-terminal of the two cysteines in the neurexin Cys-loop" - an intermolecular disulfide bond is what is likely meant here.
2. Page 5 typo 'vertebrae' > 'vertebrates'
3. Please indicate which Nrxn splice variant was used

4. I had some difficulty digesting Figure 2A, 2B as well as Figure 3G. It would be helpful if it was more clearly indicated in the Figure which protein is precipitated in these experiments.
5. Page 11, Figure 6F is referenced but this panel does not appear in Figure 6.
6. Have the authors considered a CA10 loss of function experiment to determine effects on Nrxn-HS? This would be a strong addition to the paper.
7. Are there trans-synaptic Nrxn-ligand interactions described that do not depend on HS? If so, the presence of CA10 in a neuron might boost those interactions by increasing Nrxn surface levels while at the same time reducing HS-dependent interactions.

Referee #3:

In this paper titled "CA10 Regulates Neurexin Heparan Sulfate Modification via a Direct Binding in the Secretory Pathway" Gaya, et.al. aim to identify how heparan sulfate (HS) modification on Neurexin is regulated. Previously published work showed that Neurexin can be glycosylated on the stalk region that is common to all Neurexins and HS modification of Neurexin affects ligand binding specificity. CA10 is known to be a chaperone for neurexin and overexpression of CA10 leads to an increase in neurexin surface expression, but the role of CA10 in the neurexin complex was unknown. Gaya, et.al. used a nice combination of biochemistry, NMR and mass spectrometry to show that CA10 directly binds to neurexin on either side of the serine that can be glycosylated to sterically block addition of HS. Overall, I found the paper easy to read and understand and appreciated the authors' use of appropriate controls and multiple complementary techniques to show which residues are required for CA10 binding to neurexins and that CA10 overexpression and binding to neurexin prevents addition of HS.

Major concerns:
1. Throughout the paper, the authors claim that CA10 binds neurexin in the early secretory pathway/endoplasmic reticulum, however, there are no experiments that directly test when this occurs. They do show that the first step of glycosylation is blocked, suggesting that the interaction occurs before entry into the golgi, but do not directly show this. This can be corrected with text edits that tone down the conclusion.

Minor concerns:
1. In the last sentence of the abstract, the authors state "these results suggest a mechanism for the regulation of HS-modified neurexin levels at specific synapses". While they do show a correlation between CA10 expression and neurexin surface expression in various brain regions, they do not look at neurexins at specific synapses, so this statement seemed like a bit of a reach and could be toned down.
2. In Figure 2A, the labeling of Nrxn1a(DeltaHS) is confusing since in the text they refer to this as Nrxn1a S>A. Labeling should be kept consistent between text and the figure.
3. In Figure 3A in the small chart next to the sequence mutations the authors use * and - without indicating what the symbols represent.
4. In Figure 7 it would be helpful to see surface levels of neurexin since the ligand binding is normalized to these levels, but they do not indicate how much CA10 overexpression increases surface levels of neurexin.
5. It would be helpful if the authors included a paragraph in the discussion about the known or speculated role these non-glycosylated neurexins play in cell adhesion or synapse formation, especially since they show that these neurexins do not bind classic binding partners with the same affinity as HS-Neurexin.

Authors' response to the reviewers' comments

In the following, we cite the reviewers' comments in full in *italics*, and provide our response in **bold** typeface.

Referee #1:
1. The paper would be strengthened of the function and structure of the heparan sulfate on neurexin could included. Heparan sulfate interacts with many cytokines and growth factors with clear effects on neurons.
Response: The function of the Nrxn HS is not well understood, although a model has been proposed in which the glycan chain cooperates in the protein-protein binding between Nrxn and some of its postsynaptic ligands that contain HS binding sites (see Zhang et al., *Cell* 2018; doi.org/10.1016/j.cell.2018.07.002). Our results are compatible with this model, although we cannot exclude that the Nrxn HS chain may also act by recruiting unknown cytokines and growth factors. The predicted physical length of the glycan chain indeed leaves room for multiple interactions and it may thus do both. We have added a sentence and a reference indicating the possible contribution of cytokines and growth factors in the introduction and extended the discussion, but refrained from excessive speculation.
Characterizing the composition and sequence domains of the Nrxn HS GAG chain and whether this differs from those of other HSPGs is indeed an important topic for the field. However, limitations in current techniques to read and analyze GAG sequences and requirement to purify large amounts of Nrxns from brain tissue complicate this experiment, which we believe is beyond the scope of our current paper.

2. The authors claim that the reason for elimination of heparan sulfate is that the serin motif is blocked. Another possibility is that the xylosyltransferases could be blocked. This should at least be discussed
Response: We find it unlikely that CA10 blocks xylosyltranferase activity, for the following reasons: (1) expression of CA10 does not prevent heparan sulfate to be added to the GIGG Nrxn mutant, to which CA10 cannot bind (Fig 3A-C); (2) expression of CA10 does not prevent xylosyltransferase activity on to the HSPG glypican, included now as new Figure EV2C. Thus, presence of CA10 does not block xylotransferase activity *per se*, but only to the specific neurexin serine, by binding to surrounding residues. Viewed together with the NMR experiments (see also model in figure below), we find steric hindrance to be the most likely explanation. This model is further supported by the finding that CA10 also blocks alternative GalNAc glycosylation of the same serine in HEK293 cells. Nevertheless, we cannot exclude that CA10 may inhibit xylosyltranferase activity onto CA10-bound substrates, with the same effect, and we have added this to the discussion.

3. CA10 blocks both heparan sulfate and the small HexNAc oligosaccharides. Which of these saccharides are of importance for the neurexin glyco complex
Response: The stalk region of Nrxns is rich in Ser/Thr and known to carry O-linked (HexNAc) glycosylations, presumably at multiple residues given the relatively large size shift following enzymatic removal (Ushkaryov et al., *Science* 1992; Ushkaryov et al., *JBC* 1994). These juxtamembrane glycosylations are believed to extend the stalk away from the membrane, although their functional roles have not been thoroughly investigated. The HS, on the other hand, is only added to a single serine (e.g. Nrxn1a S>A in Fig. 2A). We thus find it likely that neurexin molecules that carry the HS chain also carry a number of O-linked (HexNAc) glycosylations on nearby residues. It is not known whether the HS-modified serine, in absence of HS, instead becomes HexNAc-glycosylated, as we observed in HEK293 cells. We have unfortunately been unable to

resolve the predicted glycopeptides by mass spectrometry of mouse brain samples, which may have answered this question. Nevertheless, we believe that the HexNAc glycosylation of the otherwise HS-modified serine in the HEK293 cells could be an artifact caused by the inefficient HS biosynthesis in this cell type.
While we have not found a way to investigate the role of this specific HexNAc residue, we have added a heparinase experiment (Fig 7) to demonstrate that the main effect of CA10 in the artificial synapse formation assay depends on HS.

4. The extra bands emerging after heparinase digestion in Fig 2 should be explained. Are other heparan sulfate proteoglycan part of the complex or are they just impurities. The core size of these proteoglycans exclude syndecans glypicans and perlecan. Does these proteoglycan play any role in neurexin synapse function. complex
Response: We do not know the nature of the 3G10-reactive bands at ~200 and ~250 kDa that emerge after heparinase digestion of neuronal samples, but note that the exact same pattern has also been found by others (see Fig 1C in Zhang et al., *Cell* 2018; doi.org/10.1016/j.cell.2018.07.002). We speculate that the bands reflect some abundant heparan sulfate proteoglycan (agrin and collagen XVIII may fit in size) that bind non-specifically in the preceding immunoprecipitation step. There is no other known HSPG in Nrxn synaptic cell adhesion complexes, but we cannot exclude the exciting possibility of yet-unknown ligands. Identifying those would however be beyond the scope of the current manuscript. We have added sentences in the results section and figure legends to clarify this.

5. The functional modulation of synapses by CA10 is interesting and clear för NLG1. However the conclusion of effect on TrkC is not clearly shown in fig 7C. There is a clear read staining on the control however no red staining after treatment with CA10. Please explain your conclusion and improve the figure. More serious is that the effect can not be attributed to heparan sulfate in the present stage. Control with heparinase digestion must be performed. As pointed out in point 1 effect of heparan sulfate binding cytokines on synapse formations should be of interest.
Response: TrkC is a post-synaptic receptor that can induce formation of presynaptic specializations by recruiting pre-synaptic protein tyrosine phosphatase receptor sigma (R-PTPσ, gene name PTPRS), independent of Nrxn. Use of TrkC in this experiment serves as a control for specificity, to demonstrate that CA10 specifically affects neurexin-mediated synapse formation: we observe no effect of CA10 on TrkC-mediated synapse formation, and thus conclude that synapse formation via R-PTPσ is unaffected. The text has been edited to hopefully clarify this.

6. Do you know where the CA10- neurexin complex is formed in ER or in any of the Golgi compartment. It could however be compared with the xyloxylation of proteoglycans which occur in rough ER https://doi.org/10.3109/03008208408992780
Response: As CA10 exerts its effect on Nrxns via a direct interaction, we conclude that binding must occur prior to Nrxn xylosylation, i.e. in the ER. We apologize that this was not clearly stated and have revised the discussion.
Where xylosylation of proteoglycans occurs is not fully resolved. While early work, cited above, detected xylosyltransferases in the rough ER, subsequent papers have found most of it to reside in Golgi (Joshi et al., Cell 2018, doi.org/10.1016/j.cell.2018.01.016; Al-Jezawi et al., Am J Med Gen 2017, doi.org/10.1002/ajmg.a.38244; Schön et al., JBC 2006 doi.org/10.1074/jbc.M510690200). Nevertheless, pulse-chase experiments with radiolabeled UDP-xylose in permeabilized chondrocytes have found xylosyltransferase activity in the late ER, or perhaps in the intermediate compartment between the rough ER and *cis*-Golgi (Vertel et al., *JBC* 1993). We have modified the discussion to take this into account.

Minor points
1. The title is unclear. Do the authors mean modified neurexin or modified heparan sulfate
Response: We have revised the title to 'CA10 Regulates Neurexin Heparan Sulfate Addition via a Direct Binding in the Secretory Pathway'.

2. What does the author mean by HS modification
Response: That a heparan sulfate chain is added to the core protein (Neurexin) as a posttranslational modification. This heparan sulfate chain has been shown to be of relevance to various neurexin functions in previous publications (e.g. Zhang et. al, 2018, Cell). The word modification was chosen as it relates to "posttranslational modification", but we understand that it may bring confusion as the heparan sulfate chain itself is also modified. To avoid this conclusion, we have revised the title and the text to instead use the word "addition" when referring to neurexins. However, we have kept the term "HS-modified" when referring to the serine.

3. What is HA tag? Does it have any relation to Hyaluronan
Response: The hemagglutinin (HA) tag consists of an epitope corresponding to amino acids 98-106 of the influenza virus surface protein hemagglutinin. Due to availability of good antibodies, the HA tag is often inserted in expression vectors. We apologise for omitting to spell out the explanation and have revised the text.

4. Fig 2B. Why is HA-Nrxn1alfa+Ca10-V5 smaller than HA-Nrxn1alfa
Response: We believe that this slight apparent difference reflects an artifact caused by uneven migration of the gel front ("smiling"), but which is difficult to see due to the "empty" lanes. We do not see the same shift in replicate gels of the same experiments.

5. Where is 6F
Response: It should read 6E and this has been corrected in the text. We apologize for this mistake.

6. Figure 8. Can you really state that CA10 not affects the formation of other proteoglycans. Remove glypicans and use heparan sulfate proteoglycans. See point 4 on major suggestions.
Response: As CA10 blocks the heparan sulfate of neurexin via a direct and specific binding, we have no reason to believe that it would affect other proteoglycans. In HEK293 cells, CA10 does not block the heparan sulfate of glypican-1 (GPC1); we have included this control experiment as a new Fig EV2C. We thus believe the effect of CA10 is specific to neurexins. However, as we have not tested all HSPGs we have revised Fig 8 as suggested and toned down the discussion.

7. What does the stars indicate in some of the figures and why are the bands stronger in Figure 2 in the presence of CA10
Response: The stars indicate non-neurexin proteins of unknown identity that are immunoreactive to the 3G10 antibody (see discussion under major point #4, above). The reason they are enriched following immunoprecipitation using a CA10 antibody is not known; It may reflect co-immunoprecipitation with (non-HS) neurexin-CA10 complexes, although we cannot exclude unspecific binding to the beads and/or antisera. We have added sentences in the figure legends and results section to clarify this.

Referee #2:
1. The authors convincingly show that the intermolecular disulfide bond is not required for CA10 blocking HS-modification of Nrxn. The role of the non-covalent interaction is less clear to me. The Nrxn GIGG mutant does not bind CA10 and is HS-modified. The DIRR mutant

however also does not bind CA10 but is not HS-modified (Figure 1B, 3A and Sterky et al., PNAS 2017). How can the non-binding mutant prevent HS-modification? This should be experimentally addressed or explained.

Response: We apologize if the difference in the properties of the studied Nrxn mutants has not been properly explained. The two referred mutants (GIGG and DIRR) differ in their intrinsic capability to acquire an HS chain (see Fig 1B-C): Mutating the neurexin DILV residues to DIRR completely abolishes the HS addition also in absence of CA10, whereas the GIGG mutant retains the ability to be glycosylated. The likely explanation is that the substrate binding pocket of the xylosyltransferases prefer negatively charged residues (Glu or Asp) in the -3 and -2 positions relative to the target serine (Yu and Linhardt, *Structure* 2018; doi.org/10.1016/j.str.2018.05.011). The incorporation of positive charges – with two Arg residues in the DIRR mutant – interfere with this constraint. Indeed, docking our NMR-derived neurexin peptide structure onto the structure of xylosyltransferase 1 (Briggs and Hohenester, *Structure* 2018; doi.org/10.1016/j.str.2018.03.014) demonstrates how the Arg residues would have to be positioned in the positively charged environment in the cleft (see Figure below). Glycines in the same position are however tolerated, as indicated by the fact that the GIGG mutant becomes HS-modified.

In the experiment in which CA10 is co-expressed (Fig. 3A-B), the DIRR mutant is still

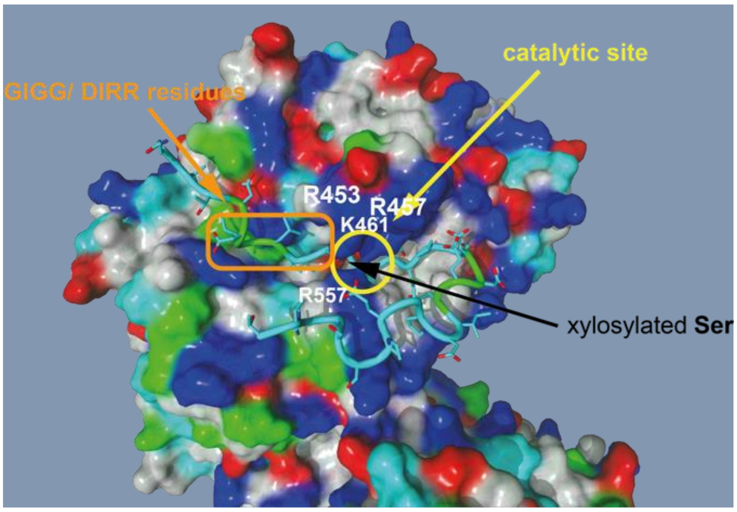

Lowest energy structure of the Nrxn1-stalk peptide from the NMR ensemble superimposed on the structure of substrate-bound xylosyltransferase 1 (XT1; substrate omitted for clarity), obtained from PDB 6ejd (Briggs and Hohenester, *Structure* 2018). Peptide structure is shown in ribbon style with sidechains shown as sticks (atom color: cyan – C, blue – N, red – O, green – S). The protein is shown in surface representation (molecular surface) and residues are colored according to their physicochemical properties (blue – basic, red – acidic, cyan, green – polar, gray – hydrophobic).

not HS-modified and here serves as a negative control. However, the GIGG mutant is HS-modified (Figure 1B-C), demonstrating that these mutations are tolerated by the xylosyltransferases. However, this mutant does not bind Nrxn (Sterky et al., PNAS 2017) and thus can acquire HS also in presence of CA10 (Figure 3C). This result demonstrates that a direct interaction between CA10 and Nrxn is necessary to prevent addition of the HS. We have revised the text to hopefully make this clearer.

2. What was the rationale for not testing LRRTM2 in the coculture assay shown in Figure 7C and 7D? Based on the Scatchard analysis in Figure 7A, the effect of CA10 expression is expected to have a stronger effect on LRRTM2-mediated synaptogenesis than on Nlgn1. This data, even when negative, should be included. The effect of HS on LRRTM2-mediated synaptogenesis in coculture assays may vary depending on the heterologous cell line used (compare Zhang et al., Cell 2018 and Condomitti et al., Neuron 2018).

Response: We did not include LRRTM2 in our initial co-culture experiments, but have done so now and incorporated the results in a revised Fig 7. Even though there is an effect of CA10 expression on Nrxn binding to LRRTM2 (Fig 7A), there is no effect on LRRTM2-induced synapse formation (Fig 7B-C). We speculate that this apparent discrepancy may be due to the kinetics of the artificial synapse formation assay; While the neurexin HS chain is predicted to enhance neurexin binding to LRRTM2, it

is not required for the interaction (e.g. crystal structures of the neurexin-LRRTM2 complex have been solved in absence of HS). Artificial synapses formed onto LRRTM2-expressing cells may thus reach saturation also in absence of the neurexin HS, despite a slightly lower affinity. Alternatively, the mechanisms whereby the HS contributes to synapse formation may differ between the two ligands.

3. Throughout the paper, the statistics are not clearly described, which makes it difficult to interpret the data. Major points that should receive attention include 1) The authors only use student's T tests or one or two-way ANOVA's with posthoc Bonferroni tests. These tests assume normal distribution of the data and (in the case of the ANOVA's with Bonferroni tests) homogeneity of variance. However, it is unclear from the Material and Methods section if the data was tested for normality and homogeneity of variance. Violation of the assumptions for these tests may result in inflated type 1 error rates. 2) In addition, for Figure 7A and 7B, the authors use a two-way ANOVA. First, this test assumes the independent variables to be categorical, but the "Concentration" variable is continuous. An ANCOVA test should be a better solution for this data. Second, the authors do not describe any posthoc tests on these data. Therefore, it is unjustified to claim that LRRTM2 or Nlgn1 binding is different between the control and CA10 conditions. 3) In multiple cases throughout the paper, statistical tests are not described. For example, in figure 2E and 2H, T tests were used to test the non-digested conditions, but it is unclear how the heparinase conditions were tested. Though by eye the results in these conditions do not seem to differ, it is still unjustified to claim that no difference is observed here without reporting statistical tests. It is highly recommended to note exact p-values for non-significant data in the figure legends as they will support multiple results in the paper.
Response: We have thoroughly revised the statistics, and clarified this in methods, figure legends and author checklist. We would like to emphasize, however, that none of our results rely on statistical inference, except for the results of Fig 7. Instead, our conclusions derive from qualitative data, primarily from immunoblots in which specific bands are either present or absent: The accompanying quantifications serve to illustrate that we have performed the experiment multiple times with the same outcome. We have thus removed statistics from Fig 1 and EV1.
As our datasets are in general underpowered for formal tests of normality, which would require each experiment to be repeated perhaps 20 times more, we have used QQ plots to assess distribution. In cases with apparent non-normal distribution, e.g. with some data points being at or near 0, we have now instead used non-parametric Mann-Whitney tests, or removed the statistics (Fig 1/EV1). Relevant non-significant p-values have been added. For other experiments, ANOVA with Holm-Sidak's posthoc tests were used. Curve-fitting by non-linear (least squares) regressions were used to compare binding curves, resulting in significance for LRRTM2 binding, but not for Neuroligin-1. The results have been revised to account for this.
We would finally like to point out that a reason for our low n is that we consistently define n as the number of independent experiments performed (i.e. while 5-10 cells per cover slip were analyzed, results were averaged to a single data point). We believe this is more rigorous than the common practice, at least within neuroscience, to define each cell imaged as an independent n (e.g. see Zhang et al., *Cell* 2018; doi.org/10.1016/j.cell.2018.07.002).
Upon revising Fig 1 and EV1 we realized that the background normalization had been done differently than for subsequent experiments. We have corrected this for consistency, resulting in slightly different numbers in the bar graphs, but without changing the conclusions of the experiments.

Minor
1. Page 4 "This cysteine can form an intramolecular disulfide between CA10 and the N-terminal of the two cysteines in the neurexin Cys-loop" - an intermolecular disulfide bond is

what is likely meant here.
Response: The typo has been corrected.

2. Page 5 typo 'vertebrae' > 'vertebrates'
Response: The typo has been corrected.

3. Please indicate which Nrxn splice variant was used
Response: This is stated in the materials and methods section. For Nrxn1-HA: SS1- SS2- SS3+ SS4- SS5+ SS6+ and for Nrxn1b-Fc: SS4- SS5-

4. I had some difficulty digesting Figure 2A, 2B as well as Figure 3G. It would be helpful if it was more clearly indicated in the Figure which protein is precipitated in these experiments.
Response: Schematic outlines of the experiments has been included in the figures (new Fig 2A and 2C). We hope this will help readers to understand the experimental design.

5. Page 11, Figure 6F is referenced but this panel does not appear in Figure 6.
Response: We apologize for this mistake. It should read 6E and this has been corrected in the text.

6. Have the authors considered a CA10 loss of function experiment to determine effects on Nrxn-HS? This would be a strong addition to the paper.
Response: Yes. We have indeed generated CRISPR/Cas9-mediated constitutive knockouts for *Ca10* and isolated a line carrying a 61 bp deletion in exon 2. Resulting homozygous mutant mice are viable and apparently healthy. These mice show, in some experiments, a slight reduction in total neurexin levels, but without apparent change in isoform distribution. However, residual levels of Ca10 protein could be detected in these mice and follow-up experiments revealed that the genetic deletion results in *Ca10* transcripts that skip exon 2 to generate a truncated protein that carries the full CA-like domain as well as the C-terminal sequence and a partial signal sequence that appears to be sufficient to target it to the secretory pathway. Exogenous expression of this hypomorphic protein could indeed block neurexin HS. Because of this confounding factor, we do not know how to interpret the results from these mice and have decided not to include them.
We have instead raised conditional knockout mice (for exon 3 of *Ca10*). Since February we have tried to ship these mice to Gothenburg, but the covid-19 outbreak has prevented all air transport of live mice from the country of origin. Due to these unfortunate circumstances, we have decided to publish our results without knockout experiments.

7. Are there trans-synaptic Nrxn-ligand interactions described that do not depend on HS? If so, the presence of CA10 in a neuron might boost those interactions by increasing Nrxn surface levels while at the same time reducing HS-dependent interactions.
Response: It has so far not been explored if there are trans-synaptic Nrxn ligands that do not depend on HS. However, the secreted ligand cerebellin-1 (cbln1) lacks known HS binding sites and we hypothesize that this neurexin ligand does not depend on HS. Consistent with this idea, we find that recombinant cbln1, attached to antibody-coupled beads, recruits pre-synaptic assembly in cultured hippocampal neurons equally well irrespective of CA10 (new data added to Fig 7).

Referee #3:
Major concerns:
1. Throughout the paper, the authors claim that CA10 binds neurexin in the early secretory pathway/endoplasmic reticulum, however, there are no experiments that directly test when

this occurs. They do show that the first step of glycosylation is blocked, suggesting that the interaction occurs before entry into the golgi, but do not directly show this. This can be corrected with text edits that tone down the conclusion.
Response: We have not found a way to experimentally demonstrate exactly where in the secretory pathway the two proteins interact. However, our data demonstrates that CA10 exerts its effect on Nrxns via a direct interaction and that the first step of the glycosylation, the xylosilation is the one that is blocked. It has previously been described that this step occurs in the late ER, or in the intermediate compartment between the rough ER and *cis*-Golgi (Vertel et al., *JBC* 1993). We thus conclude that it must occur in the earlier ER. We have added this to point to the discussion and also acknowledge that we have not directly shown this.

Minor concerns:
1. In the last sentence of the abstract, the authors state "these results suggest a mechanism for the regulation of HS-modified neurexin levels at specific synapses". While they do show a correlation between CA10 expression and neurexin surface expression in various brain regions, they do not look at neurexins at specific synapses, so this statement seemed like a bit of a reach and could be toned down.
Response: This statement reflects a hypothesis. We have removed this claim from the abstract but kept it in the discussion.

2. In Figure 2A, the labeling of Nrxn1a(DeltaHS) is confusing since in the text they refer to this as Nrxn1a S>A. Labeling should be kept consistent between text and the figure.
Response: We agree that the inconsistent nomenclature could be confusing to the reader and have revised the labeling in the figure to match the text.

*3. In Figure 3A in the small chart next to the sequence mutations the authors use * and - without indicating what the symbols represent.*
Response: The asterisk indicates a mutant that binds non-covalently, but fails to form the strong covalent interaction as it lacks the necessary cysteine. An explanation has been added in the figure legend.
This comment also made us realize that symbols reflecting conservation by ClustalW alignments in Fig 1 and EV1 are missing, and we have included this in the corresponding figure legends.

4. In Figure 7 it would be helpful to see surface levels of neurexin since the ligand binding is normalized to these levels, but they do not indicate how much CA10 overexpression increases surface levels of neurexin.
Response: We apologize for omitting this important information. In this experiment we did not observe increased total levels of neurexin, consistent with previous findings (see Fig 7C, Sterky et al., 2017). Unfortunately, we were unable to asses surface-levels of endogenous (non-tagged) neurexin as we are not aware of any antibody that recognize an extracellular neurexin epitope and is suitable for immunocytochemistry. We have included the quantification of immune-detected neurexin in the cell-surface binding assay, presented as a new appendix figure.

5. It would be helpful if the authors included a paragraph in the discussion about the known or speculated role these non-glycosylated neurexins play in cell adhesion or synapse formation, especially since they show that these neurexins do not bind classic binding partners with the same affinity as HS-Neurexin.
Response: See point above (reviewer #2, minor point #7), addressing the same topic.

1st Revision - Editorial Decision 18th Dec 2020

Dear Prof. Sterky

Thank you for the submission of your revised manuscript to EMBO reports. We have now received the reports from former referee 1 and 2, which are copied below.

As you will see, both referees are very positive about the study and recommend publication.

Browsing through the manuscript myself, I noticed a few editorial things that we need before we can proceed with the official acceptance of your study.

- References: please use et al for studies with more than 10 authors and list the first 10 authors.

- Please describe all new findings in the abstract in present tense.

- Figure callouts:
Please add a callout to Fig. 8 wherever appropriate
Please add callouts to the individual panels of Fig. EV1 and a callout to panel EV5C

- Appendix: I realize that you have only one figure in the Appendix but we nevertheless need a title page for the Appendix with a table of content and page numbers.
- In the legend for Appendix Figure S1, please specify whether n = 4 refers to biological or technical replicates.

- I noticed in the Author Checklist that you plan to submit all data used to generate graphs to Dryad. If you deposit the data there, it might be useful to include a link to the dataset in the Data availability section of your manuscript.

- Our production/data editors have asked you to clarify several points in the figure legends (see attached document). Please incorporate these changes in your manuscript and return the revised file with tracked changes with your final manuscript submission.

- Finally, EMBO reports papers are accompanied online by A) a short (1-2 sentences) summary of the findings and their significance, B) 2-3 bullet points highlighting key results and C) a synopsis image that is 550x200-600 pixels large (width x height) in .png format. You can either show a model or key data in the synopsis image. Please note that the size is rather small and that text needs to be readable at the final size. Please send us this information along with the revised manuscript.

We look forward to seeing a final version of your manuscript as soon as possible.

Kind regards,

Martina Rembold

Martina Rembold, PhD
Senior Editor
EMBO reports

****

Referee #1:

The authors have answered and modified the manuscript EMBOR-2020-51349V2 on all my points in a very good way. For my part I therefore support publication of this manuscript.

Referee #2:

The authors have convincingly addressed all my concerns. The experimental additions and clarifications have further improved their interesting study, which is suitable for publication in EMBO Reports in my opinion.

The authors have addressed all minor editorial requests.

2nd Revision - Editorial Decision 13th Jan 2021

Prof. Fredrik Sterky
University of Gothenburg
Laboratory Medicine
Bruna Straket 16
Gothenburg 41345
Sweden

Dear Prof. Sterky,

I am very pleased to accept your manuscript for publication in the next available issue of EMBO reports. Thank you for your contribution to our journal.

At the end of this email I include important information about how to proceed. Please ensure that you take the time to read the information and complete and return the necessary forms to allow us to publish your manuscript as quickly as possible.

As part of the EMBO publication's Transparent Editorial Process, EMBO reports publishes online a Review Process File to accompany accepted manuscripts. As you are aware, this File will be published in conjunction with your paper and will include the referee reports, your point-by-point response and all pertinent correspondence relating to the manuscript.

If you do NOT want this File to be published, please inform the editorial office within 2 days, if you have not done so already, otherwise the File will be published by default [contact: emboreports@embo.org]. If you do opt out, the Review Process File link will point to the following statement: "No Review Process File is available with this article, as the authors have chosen not to make the review process public in this case."

Should you be planning a Press Release on your article, please get in contact with emboreports@wiley.com as early as possible, in order to coordinate publication and release dates.

Thank you again for your contribution to EMBO reports and congratulations on a successful publication. Please consider us again in the future for your most exciting work.

Yours sincerely,

Martina Rembold, PhD
Senior Editor
EMBO reports

****

THINGS TO DO NOW:

You will receive proofs by e-mail approximately 2-3 weeks after all relevant files have been sent to our Production Office; you should return your corrections within 2 days of receiving the proofs.

Please inform us if there is likely to be any difficulty in reaching you at the above address at that time. Failure to meet our deadlines may result in a delay of publication, or publication without your corrections.

All further communications concerning your paper should quote reference number EMBOR-2020-51349V3 and be addressed to emboreports@wiley.com.

Should you be planning a Press Release on your article, please get in contact with emboreports@wiley.com as early as possible, in order to coordinate publication and release dates.

EMBO PRESS

YOU MUST COMPLETE ALL CELLS WITH A PINK BACKGROUND

PLEASE NOTE THAT THIS CHECKLIST WILL BE PUBLISHED ALONGSIDE YOUR PAPER

Corresponding Author Name: Fredrik H Sterky
Journal Submitted to: EMBO Reports
Manuscript Number: EMBOR-2020-51349

Reporting Checklist For Life Sciences Articles (Rev. June 2017)

This checklist is used to ensure good reporting standards and to improve the reproducibility of published results. These guidelines are consistent with the Principles and Guidelines for Reporting Preclinical Research issued by the NIH in 2014. Please follow the journal's authorship guidelines in preparing your manuscript.

A- Figures
1. Data
The data shown in figures should satisfy the following conditions:

- the data were obtained and processed according to the field's best practice and are presented to reflect the results of the experiments in an accurate and unbiased manner.
- figure panels include only data points, measurements or observations that can be compared to each other in a scientifically meaningful way.
- graphs include clearly labeled error bars for independent experiments and sample sizes. Unless justified, error bars should not be shown for technical replicates.
- if n< 5, the individual data points from each experiment should be plotted and any statistical test employed should be justified
- Source Data should be included to report the data underlying graphs. Please follow the guidelines set out in the author ship guidelines on Data Presentation.

2. Captions

Each figure caption should contain the following information, for each panel where they are relevant:

- a specification of the experimental system investigated (eg cell line, species name).
- the assay(s) and method(s) used to carry out the reported observations and measurements
- an explicit mention of the biological and chemical entity(ies) that are being measured.
- an explicit mention of the biological and chemical entity(ies) that are altered/varied/perturbed in a controlled manner.
- the exact sample size (n) for each experimental group/condition, given as a number, not a range;
- a description of the sample collection allowing the reader to understand whether the samples represent technical or biological replicates (including how many animals, litters, cultures, etc.).
- a statement of how many times the experiment shown was independently replicated in the laboratory.
- definitions of statistical methods and measures:
  - common tests, such as t-test (please specify whether paired vs. unpaired), simple χ2 tests, Wilcoxon and Mann-Whitney tests, can be unambiguously identified by name only, but more complex techniques should be described in the methods section;
  - are tests one-sided or two-sided?
  - are there adjustments for multiple comparisons?
  - exact statistical test results, e.g., P values = x but not P values < x;
  - definition of 'center values' as median or average;
  - definition of error bars as s.d. or s.e.m.

Any descriptions too long for the figure legend should be included in the methods section and/or with the source data.

In the pink boxes below, please ensure that the answers to the following questions are reported in the manuscript itself. Every question should be answered. If the question is not relevant to your research, please write NA (non applicable). We encourage you to include a specific subsection in the methods section for statistics, reagents, animal models and human subjects.

USEFUL LINKS FOR COMPLETING THIS FORM

http://www.antibodypedia.com
http://1degreebio.org
http://www.equator-network.org/reporting-guidelines/improving-bioscience-research-repor
http://grants.nih.gov/grants/olaw/olaw.htm
http://www.mrc.ac.uk/Ourresearch/Ethicsresearchguidance/Useofanimals/index.htm
http://ClinicalTrials.gov
http://www.consort-statement.org
http://www.consort-statement.org/checklists/view/32-consort/66-title
http://www.equator-network.org/reporting-guidelines/reporting-recommendations-for-tumo
http://datadryad.org
http://figshare.com
http://www.ncbi.nlm.nih.gov/gap
http://www.ebi.ac.uk/ega
http://biomodels.net/
http://biomodels.net/miriam/
http://jjj.biochem.sun.ac.za
http://oba.od.nih.gov/biosecurity/biosecurity_documents.html
http://www.selectagents.gov/

B- Statistics and general methods

Please fill out these boxes (Do not worry if you cannot see all your text once you press return)

1.a. How was the sample size chosen to ensure adequate power to detect a pre-specified effect size?	Data from immunoblots are essentially qualitative, with quantifications (often n=3) primarily used to illustrate reproducibility. A higher n (4-6) of independent experiments were used in figure 7, in which conclusions rely on statistical inference. Power analysis was not performed.
1.b. For animal studies, include a statement about sample size estimate even if no statistical methods were used.	NA
2. Describe inclusion/exclusion criteria if samples or animals were excluded from the analysis. Were the criteria pre-established?	No samples were excluded.
3. Were any steps taken to minimize the effects of subjective bias when allocating animals/samples to treatment (e.g. randomization procedure)? If yes, please describe.	Animals dissected for the preparation of lysates or primary cultures were not allocated into groups.
For animal studies, include a statement about randomization even if no randomization was used.	NA
4.a. Were any steps taken to minimize the effects of subjective bias during group allocation or/and when assessing results (e.g. blinding of the investigator)? If yes please describe.	All imaging and data analysis was performed with the investigator blinded to the sample group.
4.b. For animal studies, include a statement about blinding even if no blinding was done	No blinding of animals were done (see #3).
5. For every figure, are statistical tests justified as appropriate?	Statistical tests were done by Mann-Whitney, 2-way ANOVA with Holm Sidak's tests and non-linear regression, as indicated in the figure legends. All data are reported as the mean ± standard error of the mean (SEM) with the number of independent replicates indicated in each figure legend.
Do the data meet the assumptions of the tests (e.g., normal distribution)? Describe any methods used to assess it.	Normal ditribution was estimated by assesment of the data and QQ plots. For datasets with apparent non-normal distribution, non-parametric tests were used.
Is there an estimate of variation within each group of data?	Standard error of the mean (SEM) presented for each group.

Is the variance similar between the groups that are being statistically compared?	Yes, although many dataset group sizes are too small to allow for formal testing. For datasets with very different groups (non-normal distribution), non-parametric tests were used, or (Fig 7), the negative control group with lower variance was omitted from statistical analysis.

C- Reagents

6. To show that antibodies were profiled for use in the system under study (assay and species), provide a citation, catalog number and/or clone number, supplementary information or reference to an antibody validation profile. e.g., Antibodypedia (see link list at top right), 1DegreeBio (see link list at top right).	For immunoblotting: pan-neurexin1 (rabbit polyclonal, Millipore ABN161; RRID:AB_10917110), HS stub (mouse monoclonal 3G10; Amsbio; RRID:AB_10892311), HA (mouse monoclonal HA.11, BioLegend; RRID:AB_2565006), V5 (mouse monoclonal R960-25, Thermo Fisher Scientific; RRID:AB_2556564), FLAG (mouse monoclonal M2, Sigma-Aldrich, RRID:AB_262044), actin (mouse monoclonal AC-74, Sigma-Aldrich; RRID:AB_476697) and CA10 (rabbit polyclonal HPA054825, Sigma-Aldrich; RRID:AB_2682617). For immunohistochemistry: SV2 (mouse, monoclonal SV2-c, Developmental Studies Hybridoma Bank; RRID:AB_2315387), Synapsin (rabbit, T. C. Südhof; RRID:AB_2315400), MAP2 (chicken, 2Bscientific; RRID:AB_2138173) and Neurexin-1alpha (rabbit polyclonal, Frontier Institute; RRID:AB_2571817).
7. Identify the source of cell lines and report if they were recently authenticated (e.g., by STR profiling) and tested for mycoplasma contamination.	HEK293T/17 (ATCC CRL-11268), tested negative for mycoplasma by PCR.

* for all hyperlinks, please see the table at the top right of the document

D- Animal Models

8. Report species, strain, gender, age of animals and genetic modification status where applicable. Please detail housing and husbandry conditions and the source of animals.	C57BL/6N mice (Taconic) of both sexes were maintained under conventional conditions with a 12h light and day cycle in a humidity- and temperature-controlled room.
9. For experiments involving live vertebrates, include a statement of compliance with ethical regulations and identify the committee(s) approving the experiments.	Animal husbandry and experiments were approved by an ethics committee (permit no. 134/15) and conducted in accordance with Swedish law.
10. We recommend consulting the ARRIVE guidelines (see link list at top right) (PLoS Biol. 8(6), e1000412, 2010) to ensure that other relevant aspects of animal studies are adequately reported. See author guidelines, under 'Reporting Guidelines'. See also: NIH (see link list at top right) and MRC (see link list at top right) recommendations. Please confirm compliance.	Reporting complies with ARRIVE guidelines.

E- Human Subjects

11. Identify the committee(s) approving the study protocol.	NA
12. Include a statement confirming that informed consent was obtained from all subjects and that the experiments conformed to the principles set out in the WMA Declaration of Helsinki and the Department of Health and Human Services Belmont Report.	NA
13. For publication of patient photos, include a statement confirming that consent to publish was obtained.	NA
14. Report any restrictions on the availability (and/or on the use) of human data or samples.	NA
15. Report the clinical trial registration number (at ClinicalTrials.gov or equivalent), where applicable.	NA
16. For phase II and III randomized controlled trials, please refer to the CONSORT flow diagram (see link list at top right) and submit the CONSORT checklist (see link list at top right) with your submission. See author guidelines, under 'Reporting Guidelines'. Please confirm you have submitted this list.	NA
17. For tumor marker prognostic studies, we recommend that you follow the REMARK reporting guidelines (see link list at top right). See author guidelines, under 'Reporting Guidelines'. Please confirm you have followed these guidelines.	NA

F- Data Accessibility

18: Provide a "Data Availability" section at the end of the Materials & Methods, listing the accession codes for data generated in this study and deposited in a public database (e.g. RNA-Seq data: Gene Expression Omnibus GSE39462, Proteomics data: PRIDE PXD000208 etc.) Please refer to our author guidelines for 'Data Deposition'.<br><br>Data deposition in a public repository is mandatory for:<br>a. Protein, DNA and RNA sequences<br>b. Macromolecular structures<br>c. Crystallographic data for small molecules<br>d. Functional genomics data<br>e. Proteomics and molecular interactions	A "Data Availability" section is included.
19. Deposition is strongly recommended for any datasets that are central and integral to the study; please consider the journal's data policy. If no structured public repository exists for a given data type, we encourage the provision of datasets in the manuscript as a Supplementary Document (see author guidelines under 'Expanded View' or in unstructured repositories such as Dryad (see link list at top right) or Figshare (see link list at top right).	An excel file containing raw numbers used to produce graphs is uploaded to the Dryad repository and available at https://doi.org/10.5061/dryad.z612jm69p
20. Access to human clinical and genomic datasets should be provided with as few restrictions as possible while respecting ethical obligations to the patients and relevant medical and legal issues. If practically possible and compatible with the individual consent agreement used in the study, such data should be deposited in one of the major public access-controlled repositories such as dbGAP (see link list at top right) or EGA (see link list at top right).	NA
21. Computational models that are central and integral to a study should be shared without restrictions and provided in a machine-readable form. The relevant accession numbers or links should be provided. When possible, standardized format (SBML, CellML) should be used instead of scripts (e.g. MATLAB). Authors are strongly encouraged to follow the MIRIAM guidelines (see link list at top right) and deposit their model in a public database such as Biomodels (see link list at top right) or JWS Online (see link list at top right). If computer source code is provided with the paper, it should be deposited in a public repository or included in supplementary information.	NA

G- Dual use research of concern

22. Could your study fall under dual use research restrictions? Please check biosecurity documents (see link list at top right) and list of select agents and toxins (APHIS/CDC) (see link list at top right). According to our biosecurity guidelines, provide a statement only if it could.	No.
